# A novel metagenome-derived viral RNA polymerase and its application in a cell-free expression system for metagenome screening

**DOI:** 10.1038/s41598-022-22383-x

**Published:** 2022-10-25

**Authors:** Yuchen Han, Birhanu M. Kinfu, Fabian Blombach, Gwenny Cackett, Hongli Zhang, Pablo Pérez-García, Ines Krohn, Jesper Salomon, Volkan Besirlioglu, Tayebeh Mirzaeigarakani, Ulrich Schwaneberg, Jennifer Chow, Finn Werner, Wolfgang R. Streit

**Affiliations:** 1 Department of Microbiology and Biotechnology, University of Hamburg, Ohnhorststr. 18, 22609 Hamburg, Germany; 2 Division of Biosciences, Institute of Structural and Molecular Biology, University College London, Gower Street, London, WC1E 6BT UK; 3 Novozymes A/S, Microbial Discovery, Bagsværd, Denmark; 4 Chair of Biotechnology, RWTH Aachen, Worringerweg 3, 52074 Aachen, Germany; 5 Present Address: Institute for General Microbiology, Christian-Albrechts-University, 24118 Kiel, Germany

**Keywords:** Transcription, Expression systems, High-throughput screening

## Abstract

The mining of genomes from non-cultivated microorganisms using metagenomics is a powerful tool to discover novel proteins and other valuable biomolecules. However, function-based metagenome searches are often limited by the time-consuming expression of the active proteins in various heterologous host systems. We here report the initial characterization of novel single-subunit bacteriophage RNA polymerase, EM1 RNAP, identified from a metagenome data set obtained from an elephant dung microbiome. EM1 RNAP and its promoter sequence are distantly related to T7 RNA polymerase. Using EM1 RNAP and a translation-competent *Escherichia coli* extract, we have developed an efficient medium-throughput pipeline and protocol allowing the expression of metagenome-derived genes and the production of proteins in cell-free system is sufficient for the initial testing of the predicted activities. Here, we have successfully identified and verified 12 enzymes acting on bis(2-hydroxyethyl) terephthalate (BHET) in a completely clone-free approach and proposed an in vitro high-throughput metagenomic screening method.

## Introduction

Metagenomics has played a vital role in the discovery of novel biomolecules in the last few decades. It is a powerful tool that can reach the untapped vast majority of microbial resources to answer questions about diversity and function^1–3^. The exponential increase of sequence data and rapidly evolving smart bioinformatics tools are making in silico predictions from these resources more successful. Nonetheless, the task of functional identification of biomolecules heavily relies on the successful expression and biochemical verification of target genes. Functional metagenomics, despite its potential to yield truly novel enzymes, has suffered serious challenges in protein expression. It also requires a lengthy and laborious process of generating a large number of clone libraries from environmental DNAs. In addition, a series of subcloning steps for the positive hits have to be performed to reach the target genes. Due to all these, there is an increasing non-linearity between sequence mining and getting active corresponding protein which requires a different innovative approach to address.

The main setback of functional metagenomics is associated with the host systems where transcription of foreign DNAs is greatly biased which subsequently result in low hit rate with limited diversity^4–7^. To bypass the use of cell host for expression of metagenomic DNAs, a previous study has demonstrated the use of a recombinant bacterial transcription system to generate mRNA from environmental DNAs^8^. Cell-free protein synthesis (CFPS) has been effectively used in a variety of applications including the production of difficult-to-express proteins for reasons of toxicity or solubility. In addition to diversifying the areas of application, the main focus of CFPS has been increasing the quantity and quality of synthesized protein. Applying CFPS for the rapid discovery of bioactive molecules is particularly interesting as it allows transforming functional metagenomics to host-independent systems in high-throughput platforms. The ease to customize these systems together with the suitability to perform low volume reactions creates a remarkable combination with in vitro compartmentalization of biological reactions in droplet-based technology. The success of such a combined system has been proved in enzyme engineering, protein–protein interaction studies, pharmaceutical and diagnostics applications^9,10^.

Here, we report a simple and fast protocol for the in vitro transcription and translation of metagenome-derived genes using a new RNA polymerase EM1 RNAP (the *first* RNA polymerase identified from *e*lephant/*e*nvironmental *m*icrobiome) to develop a cell-free screening pipeline for metagenome-derived resources. The current work demonstrates the combined power of in silico sequence mining tools and CFPS systems for fast, efficient, and robust screening of metagenome-derived enzymes in high and semi-high-throughput screening platforms.

## Results

The single-subunit bacteriophage RNA polymerases (ssRNAP) have distinct advantages for biotechnical and pharmaceutical applications compared to the multi-subunit RNA polymerases. They are widely used for recombinant protein expression in bacteria, as well as the in vitro transcription of mRNA and synthesis of riboprobes. However, only very few ssRNAPs are available in market, e.g., T7 RNA polymerase, which has some limitations including nonspecific RNA-templated transcription at long incubation times and the formation of double-stranded RNA regions^11–13^. Here we sought to identify a novel bacteriophage ssRNAP, on the one hand to increase the diversity of available ssRNAPs, on the other hand to meet our great demand for the setup of the cell-free metagenomic screening pipeline. To start out with, we characterized a novel RNA polymerase, designated EM1 RNAP, which proved very efficient and useful for the in vitro metagenomic screening.

### Identification of EM1 RNAP from the elephant feces microbiota

**Figure 1:**
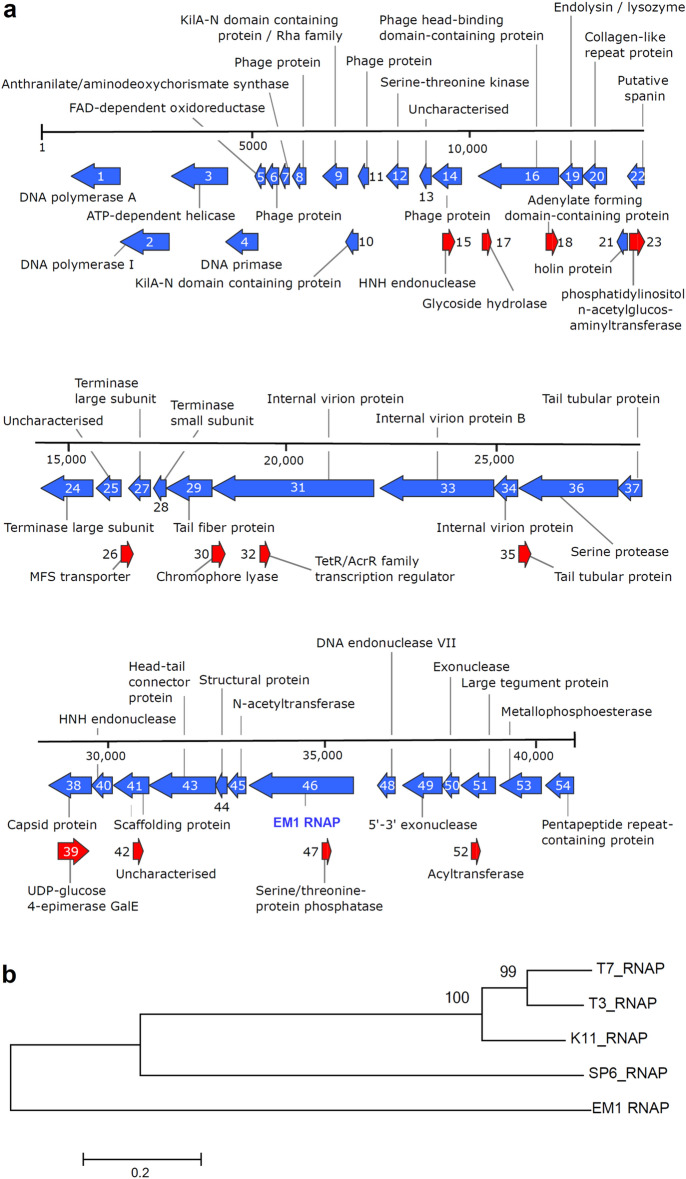
The location of EM1 RNAP and its relation with other RNA polymerases. (**a)** Annotated genome of the phage encoding the viral RNA polymerase hit (ORF46, marked as “EM1 RNAP”) assembled from a metagenome obtained from an elephant fecal microbiome in Hamburg Zoo (IMG Genome ID: 3300001598)^14^. The arrows indicate the direction of transcription. (**b)** The phylogenetic relation of five bacteriophage RNA polymerases. The phylogenetic tree was constructed with MEGA6^40^ based on the Maximum-likelihood method with 1000 bootstrap replications after multiple alignments. The percentage of bootstrap resamplings is indicated on the branches. The scale bar represents the expected number of changes per amino acids position.

About 1.31 × 10^8^ gene counts were screened from a total of 66 Gb assembled DNA within 30 metagenome datasets in IMG database using Hidden Markov Model (HMM) derived from T7, T3, and SP6 RNA polymerase sequences. A total of 107 full-length viral RNA polymerase hits have a score of more than 300. Among them a single full-length RNA polymerase hit, which is encoded on an environmental phage contig (Gene ID: EMG_100139454, Joint Genome Institute, GenBank accession number: MW765263), was obtained from elephant feces (IMG Genome ID: 3300001598)^14^. It is located on a 10 kb scaffold (IMG No. EMG_10013945) with the DNA polymerases, endonucleases and many phage proteins (Fig. 1a). We were able to assemble the 41 kb complete phage genome harboring this contig from the metagenome dataset (Fig. 1a). We detected 54 open reading frames (ORFs) of > 75 residues in length across the genome and predicted their functions (as annotated in Fig. 1a) by identifying sequence homologs via BLASTP, the majority of hits originating from other phage genomes. The phylogenetic assignment implied that it most likely belongs to the genus of the *Drulisvirus* within the family of *Autographiviridae* and the Phylum of the *Uroviricota*. The EM1 RNAP gene (ORF46) was predicted to encode for an 816-amino-acid protein. The predicted amino acid sequence carries the COG5108 domain, with homologies to mitochondrial DNA-directed RNA polymerases, Pfam 00940 RNA_pol and the Pfam RPOLN signatures and it belongs to EC:2.7.7.6—DNA-directed RNA polymerase. A more detailed data analysis showed that EM1 RNAP is similar to RNA polymerase from phages found in *Klebsiella* and other *Enterobacteria* with the highest homologies (71%) observed to a DNA-directed RNA polymerase from *Enterobacter cloacae* phage phiKDA1 (YP_009167685.1).

**Figure 2:**
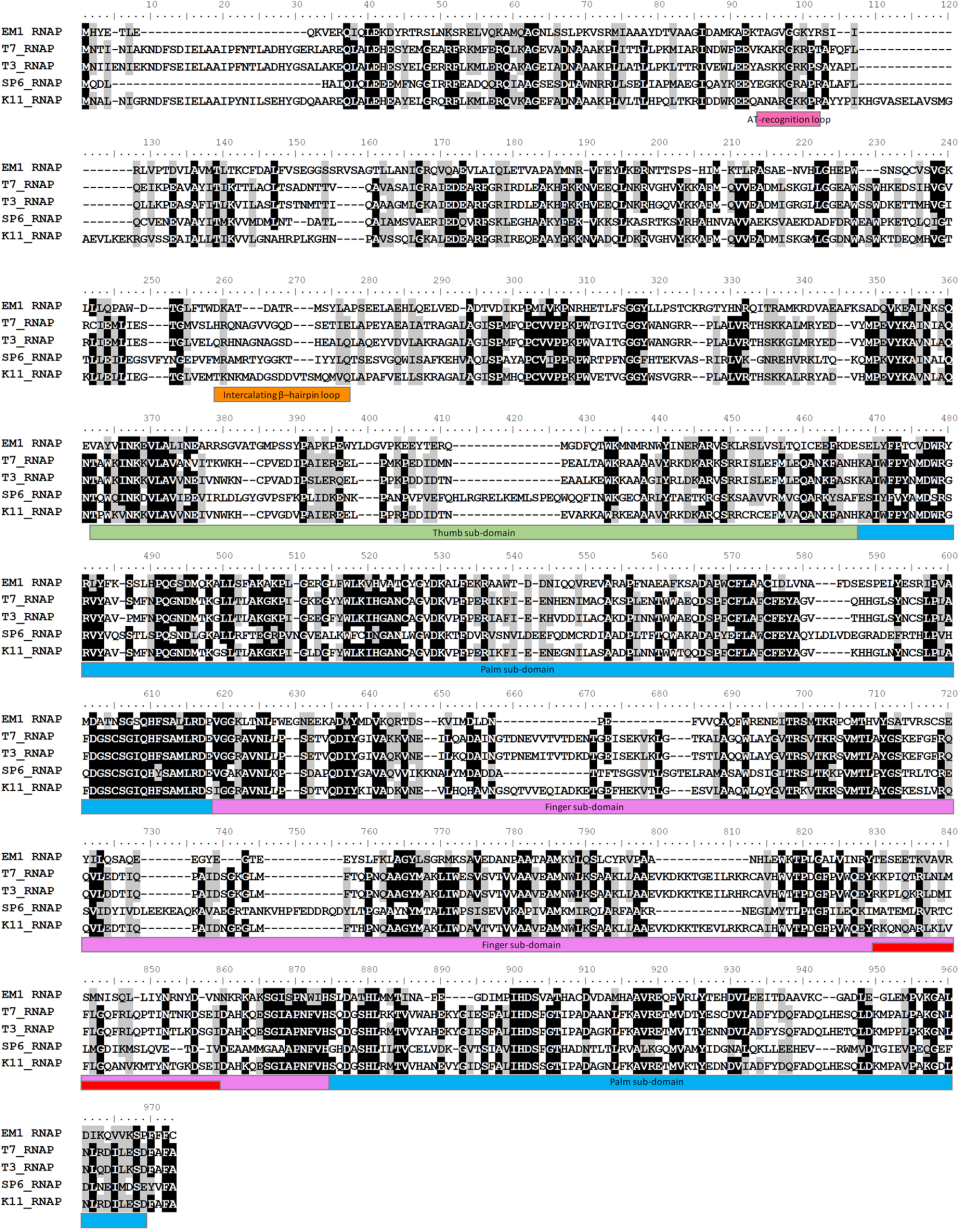
Comparison of EM1 RNAP protein sequence with other bacteriophage RNA polymerases**.** The sequence alignment was performed with T-Coffee multiple sequence alignment^31^, conserved sites of identity ≥ 80% are black-shaded and similarity ≥ 80% are grey-shaded. The color blocks under the sequences indicate the structural and functional domains of T7 RNA polymerase^16^. T7 RNA polymerase consists of an N-terminal domain and a polymerase domain. The N-terminal domain contains an AT-recognition loop (pink) and an intercalating β-hairpin loop (orange), which are important for binding the promoter and placing the template into an active site. The polymerase domain contains three sub-structural domains (light green, light blue and violet), which consist critical catalytic residues and play a key role in DNA-binding. In the finger sub-domain, a specificity loop (red) interacts with the promoter.

After codon optimization and overexpression in *E. coli* BL21 (DE3), up to 18 mg of the recombinant His-tagged EM1 RNAP protein could be purified from a liter of expression culture (Supplementary Fig. 1). EM1 RNAP is a single subunit RNA polymerase and has only 26% identity (calculated by NCBI-Blast-Global Alignment) with T7 RNA polymerase based on amino acid alignment. EM1 RNAP is divergent from other known bacteriophage RNA polymerases, such as T7 and SP6 (Fig. 1b), suggesting that it might also carry functionally divergent properties. Comparison with the crystal structure of RNAP_T7 (PDB ID: 1CEZ^15^), the predicted EM1 RNAP structure exhibits similar ‘hand’-structure including an N-terminal domain and three sub-domains, i.e., thumb-, palm- and finger-sub domains (Supplementary Fig. 2). The amino acid sequence alignment showed that the most conserved domain is the palm-subdomains (according to the T7 RNA polymerase protein sequence^16^, Fig. 2), which is important for the catalytic activity. However, the finger subdomain, which is critical for the template-binding, exhibit much lower similarity among these RNA polymerases (Fig. 2 and Supplementary Fig. 2), indicating that they might recognize different promoter sequences.

### EM1 RNAP promoter search and validation

**Figure 3:**
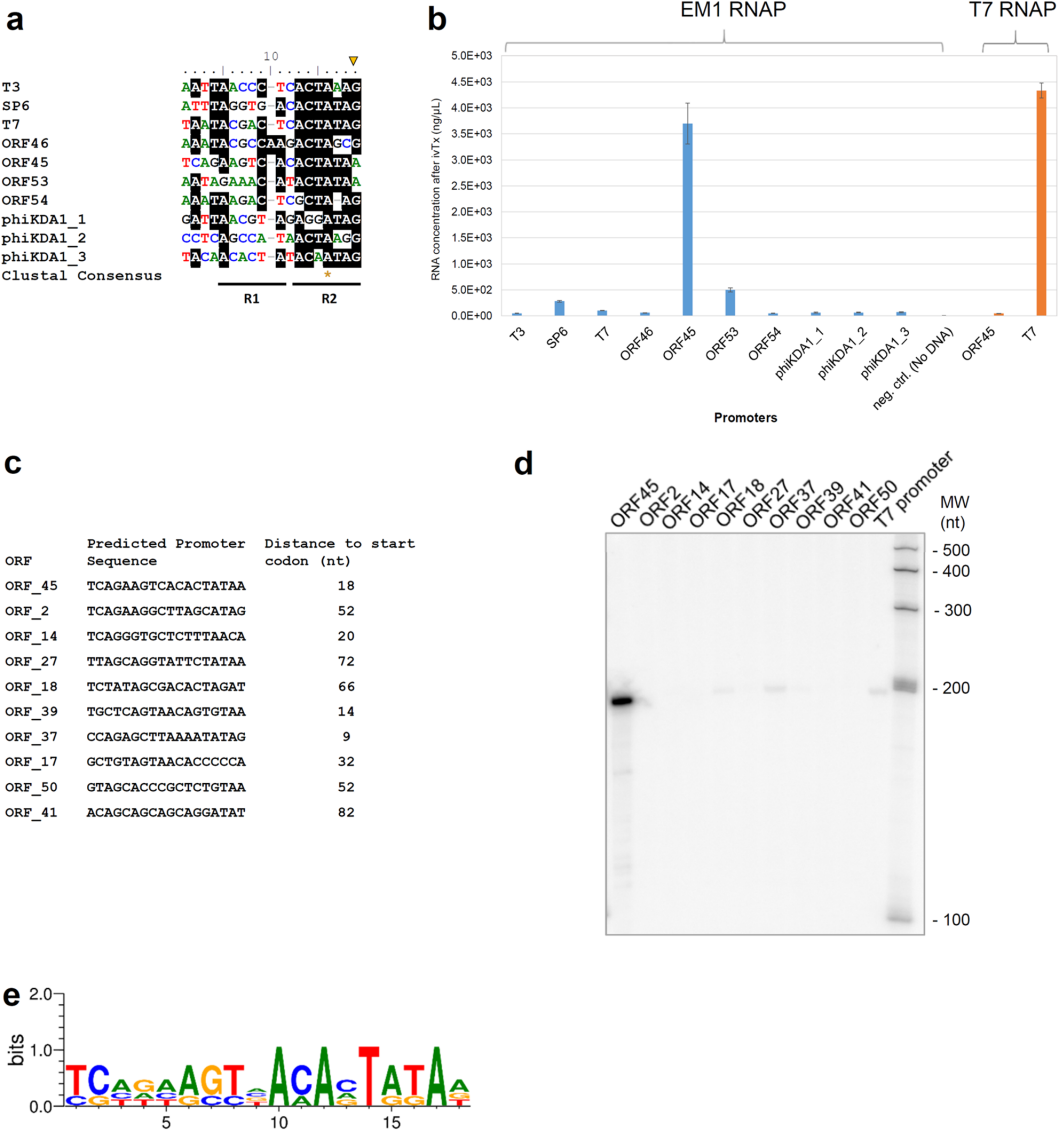
EM1 RNAP activity with different promoter sequences. (**a)** Comparison of consensus sequences from candidate promoters**.** Sequence alignments of candidate sequences and determination of consensus regions were performed to identify potential promoters (Table 2). The candidate promoter sequences were aligned with T-Coffee multiple sequence alignment, conserved sites (identity ≥ 60%) are black-shaded; an asterisk (*) indicates a single and fully conserved nucleotide; start (+ 1) is marked by a triangle; R1 is the −8 to −12 region, where promoter-specific contacts are being made in this region; R2 is the −7 to + 1 region that plays a common function in promoter binding^41^. (**b)** In vitro produced mRNA amount with EM1 RNAP and T7 RNA polymerase. Candidate promoter sequences were clone-free integrated into PET2 gene fragments using PCR primers carrying them (examples shown underlined, Table 2). Promoter ORF45, 46, 53, and 54 were derived from the metagenome, where the EM1 RNAP was found; and Promoter phiKDA1_1, 2, and 3 were derived from phage phiKDA1. In vitro transcriptions (ivTx) were performed with EM1 RNAP and T7 RNA polymerase at 37 °C. mRNA concentration was quantified with NanoDrop 2000 spectrophotometer after in vitro transcription. Error bars indicate the standard deviation from three independent experiments. (**c)** Individual promoters on the phage genome identified by the FIMO search based on ORF45 promoter. (**d)** Promoter-dependency experiment testing EM1 RNAP transcription of 200 nt RNAs in 10 µL reactions containing 0.5 µg EM1 RNAP incubated for 5 min at 37 °C. Transcripts were resolved on a denaturing gel and detected by phosphor-imagery. The original figure is presented in Supplementary Fig. 7. (**e)** Weblogo motif consensus from alignment of the four active phage promoters (ORF45, ORF18, ORF37, and ORF39) identified in (**d**).

We initially aimed to identify EM1 RNAP promoters using a priori information of the promoter sequence context of the well-characterized bacteriophages T3, T7, and SP6. The HMM logo based on the alignment of T3, T7 and SP6 promoters was used to predict EM1 RNAP candidate promoters present in 10-kb genome scaffold EMG_10013945, from which the EM1 RNAP ORF was identified. Four such candidate promoters along with various control promoters were tested in bulk in vitro transcription assays where the accumulation of mRNA is detected through UV light in a Nanodrop spectrophotometer but without discriminating transcript sizes (Fig. 3a,b). The promoter with highest activity had the sequence 5′-TCAGAAGTCACACTATAA-3′ (Fig. 3b). This promoter is located upstream of ORF45, that is predicted to encode a putative N-acetyltransferase gene (ORF45) (Fig. 1a). The ORF45 promoter has 67% identity (calculated by NCBI-Blast-Global Alignment) with the T7 promoter, however, the T7 RNA polymerase could not utilize this promoter, which demonstrates a substantial divergence in promoter sequence specificity between EM1 RNAP and T7 RNAP (Fig. 3b). Accordingly, EM1 RNAP displayed only low- (SP6, 61% identity with ORF45 promoter) or very low activity on other bacteriophage promoters including such as T7 promoter. Additional candidate promoters from the scaffold EMG_10013945 likewise displayed low- (ORF53 promoter, 68% identity with ORF45 promoter) or no activity (ORF46 promoter and ORF54 promoter) (Fig. 3b). We also tested the promoters from bacteriophage phiKDA1 (Promoter phiKDA1_1, 2, and 3, Fig. 3b) and the marine cyanophage Syn5 and *Klebsiella* phage KP34 (Data not shown), none of which were active.

In order to widen the EM1 RNAP promoter search beyond the original contig used for the identification of the EM1 RNAP gene (scaffold EMG_10013945), we used the ORF45 promoter sequence to interrogate the complete assembled phage 41 kb genome. As the ORF45 promoter displayed the highest activity of EM1 RNAP in our in vitro transcription experiments, we scanned the phage genome for similar promoter motifs in the sequence space 100 bp upstream of the predicted start codon of each ORF in the genome. Significant hits (*p* < 0.001) were identified for 10 genes (including ORF45) with 9 to 82 bp distance to the predicted start codon (Fig. 3c and Supplementary Table 1). In order to improve sensitivity and to obtain a more fine-grained picture of the mRNAs synthesized, we carried out in vitro transcription experiments using ^32^P radio-labelled RNA separated on denaturing polyacrylamide (‘sequencing’) gels, see below. This assay has a higher sensitivity, which revealed three of the additional candidate promoters to be transcriptionally active, i.e., ORF18, 37, and 39, albeit at very low levels comparable to the T7 promoter (Fig. 3d). The weblogo of these four active promoters shows increased sequence conservation at the 3′-segment of the promoter motif which both highlights the similarity as well as difference from the T7 promoter consensus (Fig. 3e).

### Characterization the activity of EM1 RNAP in in vitro transcription

We characterized the transcription activity of EM1 RNAP in two different types of assays, by testing the synthesis of the 200 nucleotide (nt) radio-labelled transcripts detected by autoradiography, and by measuring the synthesis of longer unlabelled mRNAs (1.1 knt) detected by Nanodrop spectrophotometer directly or by Qubit with RNA specific fluorescence label. This mRNA encodes PET2, a well-characterized poly(ethylene terephthalate) esterase (PETase)^17^.

Dose–response experiments revealed that the accumulation of radio-labelled 200 nt transcripts started to saturate at enzyme concentrations above 1 µg EM1 RNAP per 20 µL reaction after 5 min incubation at 37 °C (Supplementary Fig. 3a). To test the efficiency of EM1 RNAP for in vitro transcription of longer mRNAs, 0.1–25 µg of EM1 RNAP was added in the 50 µL of transcription reaction using 1 µg of DNA template consisting of the PET2 ORF sequence under the control of the ORF45 promoter. The results revealed that the highest yield of mRNA was produced by 2.5 µg to 5 µg of EM1 RNAP in this assay (Supplementary Fig. 3b). These mRNA levels are at the comparable range with the mRNA produced by T7 RNA polymerase. From here on the transcription assays were carried out using 5 µg of EM1 RNAP in a 50 µL transcription reaction volume with 1 µg DNA template, typically synthesizing between 50 and 90 µg mRNA.

**Figure 4:**
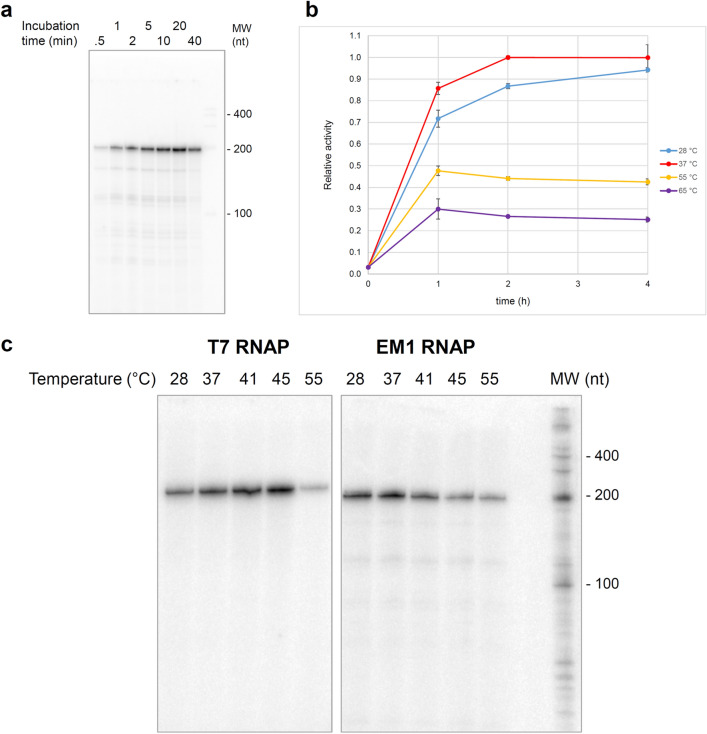
The activity of EM1 RNAP and T7 RNA polymerase at different temperatures. (**a)** Time course of 200 nt RNA synthesis by EM1 RNAP at 37 °C. 20 µL reactions containing 1.7 µg EM1 RNAP were incubated at 37 °C for the given time. Transcripts were resolved on a denaturing gel and detected by phosphor-imagery. The original figure is presented in Supplementary Fig. 8. (**b)** Relative activity of EM1 RNAP at 28 °C (blue), 37 °C (red), 55 °C (yellow), and 65 °C (purple). PET2 DNA sequence was amplified with the primers harboring ORF45 promoter and used as template for in vitro transcription with 5 µg of EM1 RNAP at the indicated temperature. mRNA concentration was quantified and the relative activity is normalized with the amount of in vitro produced mRNA at 37 °C for 2 h. Error bars indicate the standard deviation from three independent experiments. (**c)** Temperature-dependency experiment testing EM1 RNAP and T7 RNAP transcription of 200 nt RNAs in 10 µL reactions containing 0.5 µg EM1 RNAP or 20 unit T7 RNAP incubated for 5 min at 37 °C. Transcripts were resolved on a denaturing gel and detected by phosphor-imagery. The original figure is presented in Supplementary Fig. 9.

In time course experiments using 1.7 µg EM1 RNAP in 20µL reactions at 37 °C, we observed an initial near-linear increase of radio-labelled 200-nt RNA yield within the first 5 min followed by a saturation phase and even decrease in signal at 40 min, that may be caused by product inhibition caused by EM1 RNAP binding to the RNA (Fig. 4a). In comparison, the accumulation of the longer PET2 mRNA continued for much longer, with 90% of the signal reached after 60 min at 37 °C and even for 2 h at 28 °C (Fig. 4b).

In order to explore the temperature dependency of EM1 RNAP, we tested its activity in both assays over a range of temperatures. In both assays EM1 RNAP exhibits the highest activity at 37 °C (Fig. 4b,c). PET2 mRNA accumulation could also be observed at elevated temperatures of 55 °C and 65 °C plateauing after one-hour reaction time at approximately 56% and 35% compared to 37 °C (Fig. 4b). This likely reflects thermal denaturation of EM1 RNAP and was also observed in the in vitro transcription assay measuring the synthesis of radiolabeled 200-nt RNA (Fig. 4c). Importantly, we could not observe any longer RNA transcripts which demonstrates that the promoter template usage is highly specific and EM1 RNAP is not prone to RNA-templated RNA synthesis.

The influence of the ionic strength on the activity of EM1 RNAP was also tested by performing in vitro transcription in the buffers containing 10–210 mM NaCl. Compared to the normal buffer condition (10 mM NaCl), the activity of EM1 RNAP was reduced around 25% in the presence of 160 mM NaCl and around 50% by 210 mM NaCl (Supplementary Fig. 4), suggesting that EM1 RNAP is more tolerate to high salinity than T7 and SP6 RNA polymerases, whose activities are inhibited by around 50% at NaCl concentrations above 150 mM as the manufacturer described.

To find out the best long-term storage condition for EM1 RNAP, the purified protein was stored at two different temperatures (4 °C and −20 °C) in the buffer either with or without 50% glycerol. Its activity was tested by measuring the amount of in vitro produced mRNA. The results showed that EM1 RNAP is still fully active for up to 50 days in all tested storage conditions and glycerol did not influence the activity of EM1 RNAP (Supplementary Fig. 5). However, after a few days, there were some visible precipitates in the sample stored only in buffer without glycerol, indicating that glycerol might increase the solubility of EM1 RNAP. Therefore, EM1 RNAP was routinely stored in the storage buffer with 50% glycerol at −20 °C. The EM1 RNAP in this buffer with 50% glycerol was still highly active after one-year storage at −20 °C (data not shown).

### Application of EM1 RNAP in metagenomics screening with in vitro expression system

**Figure 5:**
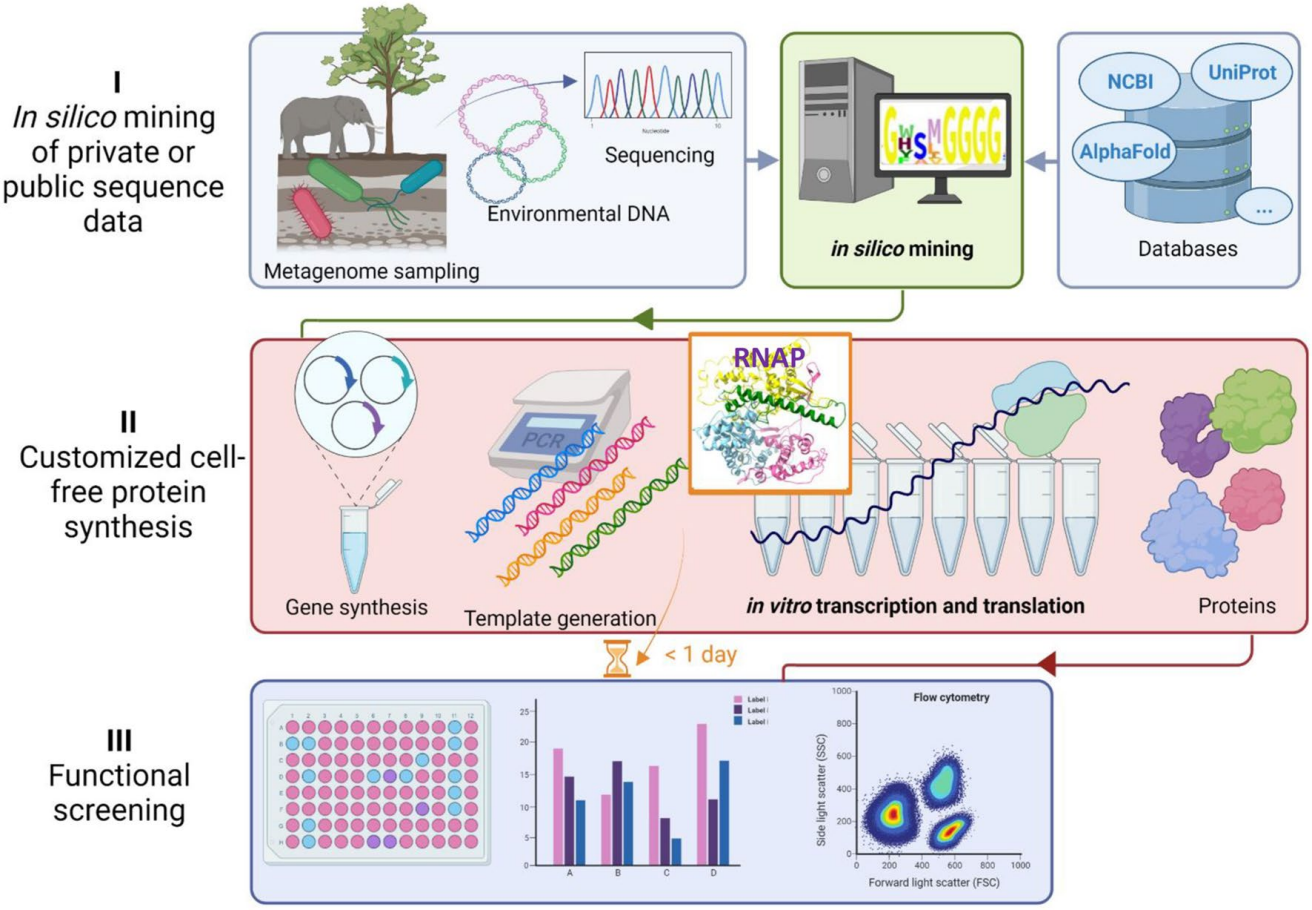
Outline of a functional metagenomic screening approach via in vitro expression system. The whole procedure can be divided into three steps: (I) in silico mining of private or public sequence data, e.g., searching for an active RNA polymerase based on the HMM profile of known RNA polymerases as presented in this work; (II) customized cell-free protein synthesis with any available RNA polymerase, e.g., in vitro expression of PETase candidates, lipase, cellulase and sfGFP as described in this work; (III) functional screening with in vitro produced proteins, e.g., different enzyme assays employed in this work. In addition to being time-efficient, this method avoids the problems often raised from overexpressing a protein in a host. Furthermore, it can be easily developed as a high-throughput technology in combination with robots.

Metagenomics screening is a very useful tool for searching new functional enzymes. While it gives fast access to genes of interests, the predicted candidate genes need to be verified with respect to their activities. Cloning and expressing each gene heterologously is time consuming and tedious if a large number of clones need to be analyzed. Therefore, we set out to develop a cell-free metagenomics screening protocol in combination of bioinformatic tools as illustrated in Fig. 5. Generally, do data mining among own metagenomic data and/or online available databases based on the HMM profile of interested proteins; then, synthesize protein in vitro with codon-optimized DNA templates; finally, apply these proteins directly for functional analyses. Here, we presented an in vitro transcription and translation system for metagenomic screening with the above in part characterized EM1 RNAP and using PETase genes as examples.

**Figure 6:**
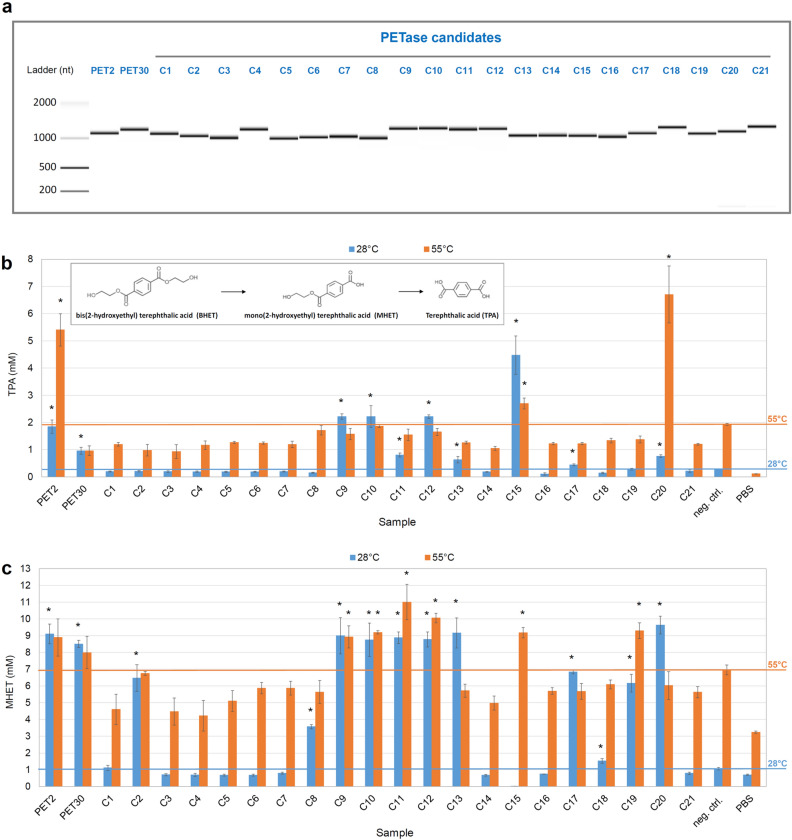
Application of EM1 RNAP in metagenomic screening of a novel PETase via in vitro expression system. (**a)** RNA gel figure of in vitro produced mRNAs from PETase candidates with EM1 RNAP. PETase candidates were identified from different metagenomes^17–19^ and their genes were synthesized in a pET vector. Then the DNA sequences were amplified with the primers harboring ORF45 promoter and used as templates for in vitro transcription with EM1 RNAP. mRNA quality was controlled with Agilent 2100 Bioanalyzer and the RNA gel figure was derived with 2100 Expert Software. The original data are presented in Supplementary Fig. 10. (**b)** The amount of TPA produced by in vitro synthesized PETase candidates using BHET as substrate at 28 °C (blue) and 55 °C (orange). (**c)** The amount of MHET produced by in vitro synthesized PETase candidates using BHET as substrate at 28 °C (blue) and 55 °C (orange). The error bars indicate the standard error from three independent experiments and the asterisks (*) indicate a significantly higher amount of TPA or MHET than the negative control with the *p*-value < 0.05. Two well-characterized PET2^17^ and PET30^19^ were shown here as controls.

For demonstration reasons, an example of a sequence mining process reported for the prediction of PETases^17,18^ was applied here. PETases genes were chosen because they are difficult to be found and only less than 30 such enzymes have been published^19^. Accordingly, 21 predicted PET-active candidate genes, together with well-characterized PET2^17^ and PET30^19^, were synthesized after codon optimization for *E. coli* system. Following the gene synthesis, a single PCR reaction was performed to introduce a specific promoter for EM1 RNAP (ORF45 promoter) upstream (5’-prime) of the target genes. PCR products were purified and transcribed into mRNA with EM1 RNAP as outlined in Materials and Methods. All 21 candidate genes and the two controls PET2 and PET30 were successfully transcribed in vitro by EM1 RNAP (Fig. 6a). All RNAs exhibited the correct size on an RNA gel figure annotated with Bioanalyzer and Agilent 2100 Expert software (Fig. 6a).

Following the mRNA synthesis and quality control, the mRNAs were translated using ribosomal complexes from *E. coli* BL21-CodonPlus (DE3)-RIL cell extracts. After translation with in vitro produced mRNA as template in our self-established cell-free system, all produced candidates were applied to an activity assay using BHET as substrate. Notably from the 23 translation assays, we observed 14 positive samples including the two positive controls. Thereby the amount of produced terephthalate (TPA) showed that these candidates exhibited different enzymatic activity concerning the degradation of BHET at the tested temperatures (Fig. 6b). PET2 is active at both 28 °C and 55 °C, while PET30 is only active at a lower temperature, which is consistent with the previous work^17,19,20^. According to the yield of TPA, C20 shows the highest activity among screened 21 candidates on the degradation of BHET at 55 °C and it is also quite active at 28 °C, but much lower than 55 °C; candidate C15 exhibits highest activity at 28 °C and mild activity at 55 °C. Candidate C9–C13, and C17 are also active at 28 °C. Besides, C2, C8, C18, and C19 did release a significant amount of mono-(2-hydroxyethyl) terephthalate (MHET) instead of TPA at 28 °C (Fig. 6c), adding another 4 active enzymes. Moreover, C9–C12 produced significantly higher amount of MHET than the negative control, indicating that they are also active at 55 °C (Fig. 6c). Thus, using this in vitro metagenomic screening method and employing EM1 RNAP 12 novel enzyme candidates have been identified. These candidate genes have not been identified as BHET-active enzymes in previous work.

Further, we asked, if other genes could be expressed based on an in vitro pipeline using EM1 RNAP. Therefore the metagenome-derived cellulase CelA2^21^, the lipase CalB^22^, and the superfolder green fluorescent protein (sfGFP^23^) were expressed using our cell-free protein expression system. In all cases, the amount of produced proteins was sufficient to perform the initial activity assay (Supplementary Fig. 6). In the case of PETase (Fig. 6) and cellulase (Supplementary Fig. 6a), the presence of translation mix or reaction components in the assay did not have significant impact on the yield. Therefore, the translation mix was directly applied to the activity assays. In the case of the expressed lipase (Supplementary Fig. 6b), the in vitro expressed lipase was first immobilized to a 96-well nickel-coated microtiter plate to select only His-tagged lipase out of the translation extract, thereby removing the background lipolytic activities from the extract. Furthermore, sfGFP has been expressed with EM1 RNAP in cell-free polymersomes instead of normal reaction tubes. Such polymersomes with sfGFP enable the development of next-generation ultrahigh-throughput functional screening of metagenomes based on flow cytometry (Supplementary Fig. 6c)^24^. All the above examples suggested that in vitro expressed proteins are active, and they exhibit detectable activity. Moreover, our in vitro expression system is a suitable tool to express a small number of proteins (20–50) in short time periods. Therefore, this in vitro expression system has been routinely applied in our lab on the metagenomic screening after data mining in order to reduce the candidate number. Until now, the longest gene what we have transcribed with EM1 RNAP is around 2 kb and its expression can be proved with Western-Blot.

## Discussion

The expression inefficiency and lengthy process of functional metagenomics have been the biggest limit for the discovery of novel biomolecules^3,25^. Developing a fast and unbiased method of activity screening for metagenomic resources is of great interest. In the classical functional metagenomics, environmental DNA is directly cloned in large insert vector carriers and shuttled to *E. coli* after phage packaging. The success of screening afterward is based on the ability of the host to actively express the foreign DNA, fold and secretion of the protein. The positive hits, apart from being at a low rate, are highly biased towards the phylogenetic origin of the host organism^25^. In industrial screening large numbers of candidate genes need to be prescreened and cloning is very time-consuming. Therefore, we have established a workflow that gives quickly access to small amounts of candidate proteins for the initial testing at small scale to verify the predicted activity by bypassing the use of host cells (Fig. 5).

The findings reported here imply that such a method can indeed be used and further developed by integrating with different technologies. Metagenomic sequences from environmental samples with or without enrichment, or sequences from databases can be taken as resources (Fig. 5). One critical advantage of this approach is the requirement of a small amount of metagenomics DNA for sequencing unlike the relatively large amount of DNA material necessary to achieve representative large clone libraries. With the sequences available, target genes are identified with iterative verification and evaluation of prediction efficiency by developing bioinformatics models. After codon-optimized and synthesized, the predicted genes for target activity are then used for template preparation for in vitro protein synthesis. Ideally, direct amplification of the target genes from the original environmental DNA should also be possible after sequencing and identification. An easy-handling and high-yield RNA polymerase, such as ssRNAPs, makes it possible to synthesize many types of RNAs under different conditions within short time. In this work, potential PETase, cellulase, lipase, and sfGFP were selected as model targets based on the nature of the protein, their activities and background interference from translation extract were analyzed, which demonstrated the high efficiency of metagenomic screening in our CFPS and the possibility of high-throughput screening with such system.

With our self-developed cell-free transcription and translation system, a novel bacteriophage RNA polymerase EM1 RNAP identified from an elephant feces microbiome metagenome was effectively used for in vitro transcription of different template DNAs. It is also interesting to note the power of the sequence mining tool where a single hit from close to 1 GB assembled metagenome DNA sequence was indeed an active RNA polymerase. This highlights the importance of carefully crafted in silico mining that can indeed guide and pinpoint effective enzyme discovery, and save a great deal of time and resources. Our reiterated promoter motif analyses started with a limited search in the 10 kb contig for EM1 RNAP promoters based on to their similarity to T3, T7 and SP6 promoters. This identified the ORF45 promoter that proved highly active in in vitro transcription assays. Using the ORF45 promoter as query, we subsequently interrogated the whole 41 kb phage genome for additional promoter candidates, well aware of that the bacteriophage-encoded RNA polymerase likely only would direct transcription from a subset of promoters of the phage genome. This second search identified a small number of additional promoters, but all of which had significantly lower activity than ORF45 promoter. Sequence comparisons between EM1 RNAP and T7 promoters show that their 3’ regions (-7 to -1 relative to the transcription start site) are more conserved compared to their 5’ regions, congruent with our overall structural understanding of bacteriophage RNAP promoter recognition and DNA binding^26^.

One of the biggest challenges in using CFPS for activity screening is the enormous amount of background interference mostly originated from the cell extract used for translation. As a result, the use of CFPS for applications such as enzyme engineering has been limited to only few enzyme types where the source organism for the translation extract (usually *E. coli*) is lacking any corresponding activity^27^. In order to tackle this, CFPS was combined with a protein capturing system using a nickel-coated microtiter plate where fusion proteins are retained in the wells while washing off the rest of translation extracts and reaction components. It was successfully demonstrated for expression and activity assay of lipase gene while avoiding high background reaching up to hundreds of folds. A limiting factor here might be the relatively high cost of the coated microtiter plate or the lengthy coating procedure. The use of anchor peptides to capture proteins onto cheaper surfaces such as plastics will be an emerging interest in this regard. Recently, this has been successfully used for applications including enzyme screening^28–30^.

To establish fast and efficient screening of metagenome-derived resources, the current platform enables finishing 1 cycle of screening from the templates within a day in contrast to weeks of intensive work in the classical approach. Here we demonstrated the use of a cell-free protein synthesis platform in its simplistic form. In order to increase the amount of protein synthesized, one can consider combining it with dialysis systems where fresh reaction components are osmotically exchanged guaranteeing continued synthesis of proteins to the level one desires.

## Materials and methods

### Bacterial strains, plasmids, and primers

Bacterial strains and plasmids as well as all primers used in this study are listed in Tables 1 and 2, respectively. *E. coli* strains for translation extract preparation and carrying gene clones were grown in LB medium (1% tryptone/peptone, 0.5% yeast extract, 0.5% NaCl) supplemented with appropriate antibiotics (100 µg/mL ampicillin or 20 µg/mL kanamycin) at 37 °C.

**Table 1:** Bacterial strains, plasmids, and constructs.

Strains, plasmids and constructs	Traits	Reference/source
**Strains**		
*E. coli* DH5α	F^−^ φ80 *lacZ*ΔM15 Δ(*lacZYA*-*argF*)U169 *recA1 endA1 hsdR*17(rk^-^, mk^+^) *phoA supE44* λ^−^ *thi*^−^1 *gyrA96 relA1*	Life Technologies (Frankfurt, Germany)
*E. coli* BL21(DE3)	F^−^ *ompT hsdS*_B_(r_B_^–^ m_B_^–^) *gal dcm* (DE3)	Novagen/Merck (Darmstadt, Germany)
*E. coli* BL21-CodonPlus (DE3)-RIL	F^–^ *ompT hsdS*_B_(r_B_^–^ m_B_^–^) *gal dcm* (DE3) *E. coli* B F^–^ *ompT hsdS*(r_B_^–^ m_B_^–^) dcm + Tet^r^ *gal* λ(DE3) *endA* Hte [*argU ileY leuW* Cam^r^]	Agilent Technologies (Waldbronn, Germany)
**Vectors and constructs**		
pET-21a( +)	5.44 kb Expression vector, *lacI*, Amp^R^, T7-promotor, C-terminal His_6_-tag coding sequence	Novagen/Merck (Darmstadt, Germany)
pET-28a( +)	5.37 kb Expression vector, *lacI*, Kan^R^, T7-promotor, N- and C-terminal His_6_-tag coding sequence	Novagen/Merck (Darmstadt, Germany)
pEX-A258::EM1 RNAP	Vector carrying synthesized gene coding for EM1 RNAP	This work
pET-28a( +)::EM1 RNAP	Expression vector carrying EM1 RNAP coding gene	This work
p1616	Vector carrying a genomic region from the archaeon *Saccharolobus solfataricus* amplified by PCR and used as template for in vitro transcription reactions	^37^

**Table 2:** List of primers used in this study.

Primer Name	Sequence (5'–3')
T3_pET	ATAAGATCTAATTAACCCTCACTAAAGGGGAATTGTGAGCGGATAAC
SP6_pET	ATAAGATCTATTTAGGTGACACTATAGGGGAATTGTGAGCGGATAAC
T7_pET	ATAAGATCTTAATACGACTCACTATAGGGGAATTGTGAGCGGATAAC
ORF46P_pET	ATAAGATCTAAATACGCCAAGACTAGCGGGGAATTGTGAGCGGATAAC
ORF45P_pET	ATAAGATCTTCAGAAGTCACACTATAAGGGAATTGTGAGCGGATAAC
ORF53P_pET	ATAAGATCTAATAGAAACATACTATAAGGGAATTGTGAGCGGATAAC
ORF54P_pET	ATAAGATCTAAATAAGACTCGCTAAGGGGAATTGTGAGCGGATAAC
phiKDA1_1_pET	ATAAGATCTGATTAACGTAGAGGATAGGGGAATTGTGAGCGGATAAC
phiKDA1_2_pET	ATAAGATCTCCTCAGCCATAACTAAGGGGGAATTGTGAGCGGATAAC
phiKDA1_3_pET	ATAAGATCTTACAACACTATACAATAGGGGAATTGTGAGCGGATAAC
pET_rev	TCCGGATATAGTTCCTC
Promoter ORF45 200 bp fusion fw	TCAGAAGTCACACTATAAGGCGTAAATTATGGGATTTTACCA
Promoter T7 200 bp fusion fw	TAATACGACTCACTATAGGGCGTAAATTATGGGATTTTACCA
200 bp rv	TTGCAGTTGTGGTGTATTTTAATCT

Underlined sequences are candidate promoters integrated into primers for template DNA preparation for transcription. All primers were synthesized at Eurofins MWG Operon (Ebersberg, Germany).

### Sequence-based mining of metagenome-based target genes

To construct a profile HMM for database mining of RNA polymerase, sequences of known viral RNA-polymerases (T3, T7, SP6) were downloaded from NCBI (accession numbers: P07659, P00573, P06221) and aligned using T-coffee 11.0.8 in accurate mode^31^. The resulting alignment was converted into a profile HMM using hmmbuild of the HMMER 3.1b2 package^32^. This model was subsequently used to screen assembled metagenomic datasets from IMG database. Only hits in complete ORFs with a score > 300 were retained. A viral RNA polymerase hit from our metagenome (IMG Genome ID: 3300001598) obtained from a feces sample of a 6-year-old elephant was used for in vitro transcription of metagenome-derived genes^14^.

The same in silico mining strategy was applied for the prediction of PETase candidates from metagenome databases^18^. 21 candidates after iterative sequence searches were selected and corresponding genes were synthesized into pET21a( +) vector after codon optimization (Biomatik, Wilmington, USA). PCR amplified DNA fragments with primers carrying promoter sequences (Table 2) were used as templates for CFPS and subsequent activity screening.

### Assembly of the phage genome

The raw reads of the Illumina sequencing were assembled using metaviralSPAdes^33^, resulting in 825 putative viral contigs, with an average length of 12.6 Kb. The phage DNA containing the RNAP was found by BLASTn. Completeness of the 40,868 bp DNA was checked with CheckV^34^, to a complete phage with 54 genes. Then the fully assembled phage genome in fasta format was imported into SnapGene (Version 6.0.2). ORFs were detected within SnapGene if > 75 residues in length. A total of 54 ORFs were identified (termed ORF1 to ORF54). The amino acid sequences of each ORF were analysed using BLASTP searches to identify any characterized homologs within the non-redundant protein sequences database, with 'Phage protein' indicating ORFs commonly detected within phage genomes, but whose function remains unknown.

### Molecular cloning, expression and purification of recombinant EM1 RNAP

The gene sequence coding for the RNA polymerase (EM1 RNAP) from elephant metagenome is codon-optimized to the *E. coli* system and synthesized as pEX-A258::EM1 RNAP construct at Eurofins (Eurofins Genomics, Ebersberg, Germany). The gene was then re-cloned into pET-28a( +) expression vector by NheI and SalI restriction-modification. After verification via sequencing, the final construct was heat-shock transformed into a chemically competent *E. coli* BL21 (DE3) expression host. The His-tagged EM1 RNAP was induced at 28 °C overnight with 0.3 mM isopropyl-b-D-thiogalactopyranoside. The purification was performed at 4 °C using Protino Ni–NTA Agarose (Macherey–Nagel, Düren, Germany) according to the manufacturer’s instruction in a lysis buffer (50 mM NaH_2_PO_4_ pH 8.0, 300 mM NaCl, 20 mM imidazole, 0.1 mM phenylmethylsulfonyl fluoride (PMSF), 1 mM dithiothreitol (DTT)). Then the agarose was washed with wash buffer (50 mM NaH_2_PO_4_ pH 8.0, 500 mM NaCl, 40 mM imidazole, 0.1 mM PMSF, 1 mM DTT). The protein was finally eluted with elution buffer (50 mM NaH_2_PO_4_ pH 8.0, 300 mM NaCl, 250 mM imidazole, 0.1 mM PMSF, 1 mM DTT). The eluted protein was dialyzed either in storage buffer (100 mM Tris–HCl pH 7.9, 100 mM KCl, 0.1 mM EDTA, 1 mM DTT, 0.1% Triton X-100, in diethylpyrocarbonate-treated H_2_O) or 2× storage buffer, which was then diluted with the same volume of glycerol. The purified protein was quantified using Bradford assay^35^ and stored either at 4 °C or −20 °C as indicated.

### EM1 RNAP promoter search and template DNA preparation

An initial promoter search was performed on the original 10-kbp metagenomic contig which contains the coding sequence for EM1 RNAP. The genome sequence of the best matching bacteriophage phiKDA1 was also scanned for additional promoter candidates. After alignments and evaluation of candidate sequences using Geneious software version 10.1.2 (Biomatters, New Zealand), selected promoter sequences were integrated into primers (Table 2) to generate PCR-amplified DNA templates for transcription. Known bacteriophage promoter sequences of T7, T3, and SP6 (Table 2) were also included as controls. After identifying ORF45 promoter as an active promoter, the promoter consensus for EM1 RNAP was reassessed by scanning the 100 bp regions upstream of the start codon of each gene (barring ORF54 being on the end of the genome) with FIMO using the known ORF45 promoter sequence 5′-TCAGAAGTCACACTATAA-3′ as query with a *p*-value cut-off of 0.001. Sequence matches were found upstream of 19 ORFs. The ten highest scoring sequences (highest FIMO score, lowest *p*-value) are shown in Fig. 3c and Supplementary Table 1.

### Preparation of translation extracts

A short and cost-effective protocol for translation extract preparation from strains of *E. coli* was pursued using the recommendations from Kwon and Jewett^36^. *E. coli* BL21-CodonPlus (DE3)-RIL (Agilent Technologies, Waldbronn, Germany) was grown in LB medium at 37 °C to mid-exponential growth phase (OD_600_ ≈ 2.0). Then the cells were harvested by centrifugation at 5000×*g* for 15 min at 4 °C. Cell pellets were washed three times with cold Buffer A (10 mM Tris–acetate pH 8.2, 14 mM magnesium acetate, 60 mM potassium glutamate, 2 mM DTT). Washed pellets were weighed, flash-frozen in liquid nitrogen and stored at −80 °C for a maximum of three days before processing.

Cell pellets were thawed the next day and suspended in cold Buffer A (1 mL of Buffer A per 1 g of wet cell mass). Cells were lysed utilizing sonication (Output ctrl 0.5, duty cycle 50%) on ice. Cells were sonicated for six periods of 1 min each, with 1 min interval between each sonication. Then the cell debris and insoluble materials were removed by centrifugation at 4 °C with a speed of 16,000×*g* twice, each time for 20 min. Finally, the cell extracts were aliquoted to 60 μL in RNase-free PCR tubes, flash-frozen in liquid nitrogen and immediately stored at −80 °C.

### In vitro transcription assays for short transcripts with radio-labelling

Templates for the synthesis of short 200 nt transcripts were generated by PCR fusing ORF45 promoter (for EM1 RNAP) or the T7 consensus promoter to a 200 bp sequence using primers Promoter ORF45 200 bp fusion fw or Promoter T7 200 bp fusion fw (Table 2), respectively with the 200 bp rv primer (Table 2) and plasmid p1616 as template^37^.

In vitro transcription reactions contained 5 ng/µL DNA template and 0.5 mM of each ribonucleotide triphosphates (rATP, rCTP, rGTP, rUTP, Promega, Mannheim, Germany) supplemented with trace amounts of [α-^32^P]-UTP (Hartmann Analytics, Germany), 1 U/µL of RiboLock RNase inhibitor (Thermo Scientific, Darmstadt, Germany), 2 mM of spermidine, 10 mM of DTT, 6 mM MgCl_2_, 10 mM of NaCl, 40 mM of Tris–HCl buffer (pH 8.0). For transcription reactions with T7 RNA polymerase, the buffer was replaced by the one supplied by the manufacturer (Thermo Scientific).

Reactions were carried out with the indicated incubation times and temperatures, reaction volumes and RNA polymerase amount, and stopped by the addition of 1 vol. 2 × formamide loading dye before. After 5 min denaturation at 95 °C, 5 µL of each sample was resolved on denaturing gels and transcripts were detected by phosphorimagery on a Typhoon FLA 9500 scanner (GE Lifesciences). All images are full-size exports of the phosphorstorage screens and only cropped at the sides of the gels to remove non-related lanes.

### Non-radioactive in vitro transcription and in vitro translation

To synthesize the target protein in a cell-free system, in vitro transcription and translation reactions were performed in the un-coupled mode.

Independent from the translation reaction, in vitro transcription reaction was composed of 0.9 mM of each ribonucleotide triphosphates (rATP, rCTP, rGTP, rUTP, Promega), 0.1 U/µL of inorganic pyrophosphatase (NEB, Frankfurt am Main, Germany), 1 U/µL of RiboLock RNase inhibitor, 2 mM of spermidine, 10 mM of DTT, 6 mM MgCl_2_, 10 mM of NaCl mixed in 40 mM of Tris–HCl buffer (pH 7.8). A standard concentration of 1 µg PCR amplified linear DNA template was used in a 50 µL transcription reaction. The transcription mixture was incubated at 37 °C for 2 h followed by DNaseI (2 U/μg template DNA, Thermo Scientific) treatment for further 15 min at 37 °C. Synthesized mRNA was then purified using RNA Clean & Concentrator-5 Kit from ZYMO Research (Freiburg, Germany). The quality and quantity of the mRNA were determined by NanoDrop 2000 Spectrophotometer (Thermo Scientific). The size of in vitro synthesized RNAs was checked with Agilent RNA 6000 Pico chip in the Agilent 2100 Bioanalyzer (Agilent Technologies) according to the manufacturer’s instruction. The same reaction mixture was also used to characterize the activity of EM1 RNAP and T7 RNA polymerase for evaluating promoter candidates. When temperature stability experiments of RNAPs were performed, the DNaseI treatment was skipped. At each time point mRNAs were immediately frozen at −80 °C. The purified mRNAs were quantified with Qubit RNA HS Assay Kit in the Qubit 3.0 fluorometer (ThermoFisher Scientific, Darmstadt, Germany).

To synthesize the target protein, in vitro translation was performed with in vitro produced mRNA as template. A standard reaction mixture was composed of 20 mM of each essential amino acids, 1.2 U/µL of RiboLock RNase inhibitor, 0.35 mM of adenosine 5′-triphosphate (ATP), 0.33 mM of Nicotinamide adenine dinucleotide (NAD), 34 µg/mL folinic acid, 130 mM potassium glutamate, 10 mM ammonium acetate, 12 mM magnesium glutamate, 1.5 mM spermidine, 1 mM putrescine, 4 mM sodium oxalate and 0.27 mM coenzyme A (sodium salt hydrate), 23% (v/v) of translation extract with total volume of 60 µL. The reaction was incubated at 37 °C for 4 h followed by functional analysis.

### Functional enzyme assay with BHET

Most of PETases can break the ester bond of BHET to produce TPA and ethylene glycol (EG) with MHET as intermediate product^17,18,38^. Therefore, BHET is often selected as the representative substance to study the activity of PETase^17,39^. In this study, BHET and in vitro expressed PETase candidates were incubated in phosphate-buffered saline (PBS, Carl Roth, Karlsruhe, Germany) at indicated temperatures for 4 days. The yields of TPA and MHET were quantified with UltiMate 3000 UHPLC system from Thermo Scientific with a Triart C18 column (YMC Europe GmbH, Dinslaken, Germany) and a VWD-3400 detector (Thermo Scientific)^19^. The data were analyzed with Compass HyStar software package from Bruker (Billerica, MA, USA).

## Supplementary Information

Supplementary Information.

## Data Availability

The DNA and amino acid sequences of EM1 RNAP were deposited at GenBank under the accession number MW765263 and also listed in Supplementary Data. Metagenome of the adult elephant feces was available from the IMG/M ER web page of the US Department of Energy Joint Genome Institute (IMG Genome ID: 3300001598, https://img.jgi.doe.gov/cgi-bin/m/main.cgi?section=TaxonDetail&page=taxonDetail&taxon_oid=3300001598)^14^.
