# Supplementary material for: A novel metagenome-derived viral RNA polymerase and its application in a cell-free expression system for metagenome screening
Source: Sci Rep. 2022 Oct 25;12:17882. doi: 10.1038/s41598-022-22383-x (PMC9596486; doi:10.1038/s41598-022-22383-x)
Supplement: Supplementary file 1 — Supplementary Information. [file 41598_2022_22383_MOESM1_ESM.pdf]

Supplementary Material

A novel metagenome-derived viral RNA polymerase and its application in a cell-free expression system for metagenome screening

Yuchen Han, Birhanu M. Kinfu, Fabian Blombach, Gwenny Cackett, Hongli Zhang, Pablo Pérez-García, Ines Krohn, Jesper Salomon, Volkan Besirlioglu, Tayebeh Mirzaeigarakani, Ulrich Schwaneberg, Jennifer Chow, Finn Werner, and Wolfgang R. Streit

1 Supplementary Data

1.1 Method - *in vitro* translation in polymersomes and flow cytometer analysis

Polymersome generation for *in vitro* protein synthesis based on double emulsion templates [1, 2] and double emulsion generation by extrusion [3] were reported previously. Briefly, double emulsion templates were generated by utilizing a mini extruder (Avanti, Polar Lipids, Inc., Alabaster, USA) by adding 200 µl of a mixture of chloroform and hexane at a volume ratio of 38:62, containing 1 mg/mL poly(butadiene)-b-poly(ethylene oxide) (PBD-b-PEO; MW: 1200 and 600, respectively) to 100 µl *in vitro* translation reaction mixture and pushing in total two times through a 10 µm PTFE membrane (Mitex PTFE hydrophobic membrane, Merck, Darmstadt, Germany) with the extruder. The (w/o) emulsion sample containing the *in vitro* translation reaction mixture and mRNA template of sfGFP was collected in 700 µL outer aqueous phase 0.1 M NaCl containing 10 wt% poly(vinyl alcohol) (PVA, MW 13000 – 23000, 87-89 % hydrolyzed) and was subsequently encapsulated in double emulsions by pushing it through a 12 µm Whatman membrane (Whatman Nuclepore Track-Etched Membranes, Sigma-Aldrich Biochemie GmbH, Hamburg, Germany) once. Polydisperse double emulsions encapsulating the *in vitro* translation reaction were transferred into a glass vial containing 0.15 M NaCl followed by polymersome formation and incubation at 37°C for 4 hours. After, *in vitro* translation of sfGFP in polymersomes were labeled with Nile Red and analyzed by fluorescence microscopy (BX51, Olympus, Tokyo, Japan). The emulsions were filtered through 50 µm filters (CellTrics, Sysmex Partec GmbH, Görlitz, Germany) and analyzed with BD Influx cell sorter (Becton Dickinson Biosciences, Erembodegem, Belgium) as well as microscopy. Analysis was performed according to the forward scatter and to the sfGFP fluorescence intensity (λ_{ex} 485 nm, λ_{em} 510 nm) after 488 nm excitation with a fluorescence emission detection of 530± 20nm (530/40(488)). The flow cytometer was operated at 5000 events^{-1} with a 100 µm nozzle and PBS (pH 7.4, 1.06 mM KH_2PO_4, 2.97 mM Na_2HPO_4, and 155.17 mM NaCl) as sheath fluid at.

1.2 DNA and amino acid sequences of EM1 RNAP

1.2.1 Original DNA sequence of EM1 RNAP

>*EM1 RNAP* from metagenome

```
ATGCATTACGAAACATTAGAACAGAAAGTTGAACGTCAGATTCAGTTAGAGAAGGATTACCGTACACGCAGCTT
GAACAAGTCGCGTGAGCTTGTACAGAAAGCTATGCAAGCTGGTAACCTGTCCAGCCTCCCTAAGGTGTCACGTA
TGATTGCAGCTGCTTACGATACAGTAGCAGCAGGTATCGACGCCATGAAGGCAGAGAAGACAGCAGGCGTGGGC
GGTAAGTACCGCAGCATTATCCGCCTTGTCCCTACTGATGTACTGGCAGTGATGACTTTAACAAAATGCTTTGA
TGCCCTCTTTGTATCTGAAGGTGGAAGTTCTCGTGTAAGCGCAGGTACTTTACTTGCTAATATCGGTCGTCAGG
TTCAGGCCGAAGTGCTGGCTATCCAACTGGAAACAGTAGCACCGGCTTATATGAACCGTGTATTCGAATACTTG
AAAGAACGTAATACTACTAGCCCATCGCACATCATGAAGACACTGCGAGCTTCCGCAGAGAACGTGCACTTAGG
GCATGAGCCGTGGAGTAACTCGCAGTGTGTATCCGTAGGCAAGCTGCTTCTCCAGCCAGCTTGGGATACAGGGC
TGTTCACGTGGGATAAAGCTACAGACGCTACACGCATGAGCTATCTGGCTCCTAGCGAGGAGCTTGCAGAGCAT
TTGCAAGAGCTGGTAGAAGACGCAGATACTGTGGACATTAAACCGCCAATGCTGGTTAAGCCTAACCGTCATGA
AACCCTGTTCTCCGGCGGGTACTTGCTACCTAGTACTTGCAAGCGCGGTACTTATCACAACCGTCAGATTACTC
GCGCTATGAAACGTGATGTAGCTGAAGCATTTAAATCAGCCGACCAAGTGAAGGAAGCTCTCAATAAATCGCAG
GAAGTGGCTTATGTAATTAACAAGGAAGTGCTGGCATTAATTAATGAAGCGCGCCGCAGTGGCGTTGCTACTGG
TATGCCTAGCTCCTATCCAGCACCTAAGCCTGAGTGGTACTTAGACGGCGTACCTAAAGAAGAGTATACCGAAC
GTCAGATGGGCGACTTCCAGACTTGGAAGATGAACATGCGTAACTGGTACATCAACGAACGTGCACGGGTAAGC
AAGCTGCGTTCGCTTGTATCCTTAACCCAGATTTGTGAAGAGTTTAAAGATGAGTCAGAGTTGTACTTCCCGAC
CTGCGTTGACTGGCGTTACCGCCTGTACTTCAAGTCCAGTCTGCATCCTCAAGGTTCTGACATGCAGAAGGCTT
TACTCTCCTTCGCCAAAGCTAAACCTCTCGGGGAGCGCGGTTTATTCTGGCTTAAGGTGCACGTCGCTACTTGC
TACGGTTATGACAAAGCCTTATTCGAGAAGCGTGCAGCTTGGACTGATGATAATATCCAGCAAGTTCGGGAAGT
TGCACGGGCGCCGTTTAATGCAGAGGCTTTTAAATCAGCAGATGCACCGTGGTGTTTTCTGGCTGCCTGCATCG
ACCTCGTTAACGCTTTTGATTCTGAAAGCCCAGAGCTTTACGAATCGCGGATTCCGGTGGCTATGGATGCGACC
AACTCCGGTAGCCAGCACTTCAGCGCACTGCTGCGTGACCCAGTAGGTGGTAAACTTACCAACCTGTTCTGGGA
AGGTAATGAAGAGAAGGCGGATATGTACATGGATGTTAAGCAGCGTACTGACTCTAAAGTGATTATGGACTTAG
ATAATCCTGAGTTCGTAGTGCAGGCTCAGTTCTGGCGTGAGAATGAAATCACCCGCAGCATGACCAAGCGTCCT
TGCATGACACATGTTTATAGTGCAACCGTACGTTCATGCTCTGAGTACATCCTCCAATCAGCACAGGAAGAAGG
ATACGAAGGAACTGAAGAGTACAGCCTGTTCAAGCTCGCTGGCTATCTAAGCGGACGTATGAAGTCTGCGGTAG
AAGATGCCAATCCAGCAGCTACAGCAGCAATGAAGTACTTGCAGAGCTTGTGCTACCGTGTACCAGCAGCTAAC
CACTTGGAATGGAAGACACCGCTAGGTGCGCTGGTTATCAACCGTTATACTGAATCTGAAGAAACTAAAGTTGC
TGTACGCAGCATGAATATTTCTCAGCTTCTTATCTATAACCGCAACTATGATGTAAACAATAAGCGTAAAGCTA
AGTCAGGAATCTCTCCTAACTGGATTCACAGTTTAGACGCAACTCACCTGATGATGACTATCAATGCATTTGAA
GGGGACATTATGCCTATCCACGATTCAGTAGCTACACATGCCTGCGACGTAGATGCAATGCACGCAGCAGTACG
TGAACAGTTCGTACGCCTTTACACCGAGCATGATGTACTTGAAGAGATTACAGACGCTGCTGTTAAATGCGGCG
CTGATTTAGAAGGACTGGAGATGCCTGTTAAAGGTGCTCTGGATATTAAGCAAGTAGTAAAATCACCGTTCTTC
TTCTGCTGA
```

1.2.2 Codon-optimized DNA sequence of EM1 RNAP

>*EM1 RNAP* (codon-optimized)

ATGCATTACGAAACGCTGGAACAGAAAGTCGAACGCCAGATTCAACTGGAAAAGGACTATCGCACACGTTCCTT
GAATAAGTCCCGTGAACTCGTGCAGAAAGCGATGCAAGCGGGCAATCTGAGCTCTCTGCCCAAGGTTTCGCGTA
TGATTGCCGCCGCGTATGATACGGTAGCGGCCGGTATTGACGCTATGAAAGCGGAGAAAACGGCCGGTGTGGGC
GGTAAGTATCGGTCGATTATTCGCCTGGTACCGACTGATGTCTTAGCCGTGATGACCCTGACGAAATGCTTTGA
CGCCCTCTTTGTCTCTGAAGGCGGGTCATCCCGCGTTAGTGCAGGGACTCTGTTGGCGAACATTGGGCGCCAAG
TTCAAGCCGAAGTTTTGGCGATCCAGCTGGAAACCGTTGCACCAGCCTACATGAATCGCGTATTTGAGTACCTG
AAAGAACGCAATACCACTTCACCATCACACATCATGAAAACCCTGCGTGCCAGTGCCGAAAACGTACATCTGGG
CCATGAACCGTGGAGCAACAGTCAATGCGTGAGCGTCGGCAAACTGTTGCTGCAACCGGCCTGGGATACCGGCT
TATTTACGTGGGATAAAGCGACCGATGCCACGCGTATGTCGTACCTGGCACCGTCTGAGGAACTCGCGGAACAC
CTCCAAGAGCTGGTGGAAGATGCGGATACCGTTGATATCAAGCCGCCGATGTTAGTTAAACCTAATCGCCACGA
AACCTTATTCAGCGGAGGGTATCTTCTTCCGAGCACCTGTAAACGTGGCACTTACCACAATCGCCAGATTACAC
GCGCCATGAAACGCGACGTGGCGGAGGCTTTTAAAAGTGCGGATCAGGTCAAAGAAGCCCTGAATAAGTCGCAA
GAAGTGGCATACGTGATTAACAAAGAGGTGCTGGCGCTGATCAACGAAGCTCGCCGGTCAGGTGTCGCGACCGG
TATGCCGAGCTCGTATCCAGCCCCTAAACCGGAGTGGTATCTTGATGGTGTTCCCAAAGAGGAATATACGGAAC
GCCAGATGGGCGATTTCCAGACGTGGAAAATGAACATGCGCAATTGGTACATTAATGAACGTGCACGTGTGTCT
AAACTGCGCAGCCTTGTCTCTCTGACGCAGATTTGCGAAGAATTTAAAGATGAAAGCGAATTGTACTTCCCGAC
ATGTGTAGACTGGCGTTACCGTCTGTATTTCAAAAGTTCGCTTCATCCTCAGGGGTCGGATATGCAGAAAGCCC
TGCTGTCCTTTGCAAAGGCGAAACCACTCGGTGAGCGTGGACTGTTCTGGCTGAAAGTTCATGTAGCGACTTGC
TATGGTTATGATAAGGCTCTGTTTGAAAAGCGCGCAGCTTGGACCGACGATAACATCCAACAAGTACGCGAAGT
CGCACGTGCTCCGTTTAACGCGGAAGCGTTCAAATCCGCTGATGCACCGTGGTGTTTCTTAGCGGCGTGTATCG
ACTTGGTGAACGCATTTGACTCAGAAAGCCCGGAACTGTACGAATCTCGTATTCCCGTCGCGATGGATGCCACG
AACTCGGGTAGTCAGCACTTCTCCGCATTATTGCGTGATCCGGTGGGCGGAAAACTGACCAACCTGTTTTGGGA
GGGCAATGAGGAGAAAGCAGATATGTATATGGACGTCAAACAGCGTACAGACAGCAAGGTGATTATGGACTTGG
ACAACCCCGAGTTTGTGGTTCAAGCCCAGTTTTGGCGTGAAAATGAGATTACCCGCAGTATGACCAAACGCCCG
TGTATGACGCACGTGTACAGTGCCACCGTGCGTAGCTGCTCGGAATACATCCTGCAGAGTGCACAGGAGGAGGG
GTATGAAGGCACCGAGGAATACTCCCTGTTCAAATTAGCCGGTTATTTATCTGGCCGTATGAAAAGCGCGGTGG
AGGATGCGAATCCAGCCGCTACAGCAGCTATGAAATATCTCCAGAGCCTGTGCTATCGCGTTCCGGCAGCTAAC
CATCTTGAGTGGAAAACCCCTCTGGGTGCTTTGGTCATCAATCGCTATACAGAAAGCGAGGAAACTAAAGTTGC
CGTGCGGTCTATGAACATCTCCCAGCTCCTGATCTACAACCGCAACTATGATGTGAATAACAAGCGCAAAGCGA
AGTCAGGCATTTCCCCTAACTGGATTCATAGCCTCGACGCAACGCATCTGATGATGACCATTAATGCGTTTGAA
GGAGACATCATGCCGATCCACGATAGCGTGGCGACTCATGCGTGCGATGTGGATGCTATGCATGCAGCGGTACG
CGAACAGTTCGTTCGGCTGTATACCGAACACGACGTTCTGGAAGAAATTACCGATGCAGCGGTAAAATGTGGTG
CTGACCTGGAAGGCTTAGAAATGCCAGTTAAAGGCGCGCTTGATATCAAACAGGTGGTCAAATCACCGTTCTTC
TTTTGCTAA

1.2.3 Amino acid sequence of EM1 RNAP

>EM1 RNAP

MHYETLEQKVERQIQLEKDYRTRSLNKSRELVQKAMQAGNLSSLPKVSRMIAAAYDTVAAGIDAMKAEKTAGVG
GKYRSIIRLVPTDVLAVMTLTKCFDALFVSEGGSSRVSAGTLLANIGRQVQAEVLAIQLETVAPAYMNRVFEYL
KERNTTSPSHIMKTLRASAENVHLGHEPWSNSQCVSVGKLLLQPAWDTGLFTWDKATDATRMSYLAPSEELAEH
LQELVEDADTVDIKPPMLVKPNRHETLFSGGYLLPSTCKRGTYHNRQITRAMKRDVAEAFKSADQVKEALNKSQ
EVAYVINKEVLALINEARRSGVATGMPSSYPAPKPEWYLDGVPKEEYTERQMGDFQTWKMNMRNWYINERARVS
KLRSLVSLTQICEEFKDESELYFPTCVDWRYRLYFKSSLHPQGSDMQKALLSFAKAKPLGERGLFWLKVHVATC

YGYDKALFEKRAAWTDDNIQQVREVARAPFNAEAFKSADAPWCFLAACIDLVNAFDSESPELYESRIPVAMDATNSGSQHFSALLRDPVGGKLTNLFWEGNEEKADMYMDVKQRTDSKVIMDLDNPEFVVQAQFWRENEITRSMTKRPCMTHVYSATVRSCSEYILQSAQEEGYEGTEEYSLFKLAGYLSGRMKSAVEDANPAATAAMKYLQSLCYRVPAANHLEWKTPLGALVINRYTESEETKVAVRSMNISQLLIYNRNYDVNNKRKAKSGISPNWIHSLDATHLMMTINAFEGDIMPIHDSVATHACDVDAMHAAVREQFVRLYTEHDVLEEITDAAVKCGADLEGLEMPVKGALDIKQVVKSPFFFC

2 Supplementary Figures and Tables

2.1 Supplementary Figures

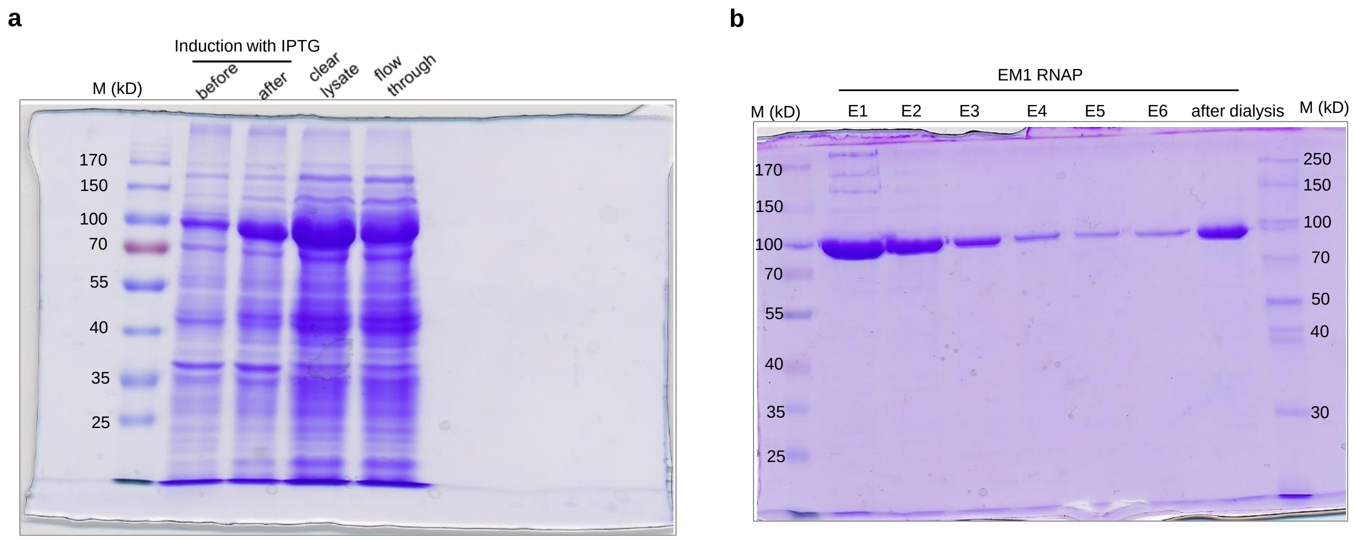

Supplementary Fig. 1 Overexpression and purification of EM1 RNAP.

EM1 RNAP was overexpressed in a vector pET28a(+) with *E. coli* BL21(DE3) as host.

a The protein expression was induced by 0.3 mM IPTG. The cell extracts before and after induction were normalized to OD600.

b The protein was purified with Ni-NTA agarose, eluted with the elution buffer containing imidazole (E1 - E6), dialyzed with storage buffer and visualized in 10 % SDS-polyacrylamide gel after Coomassie blue staining.

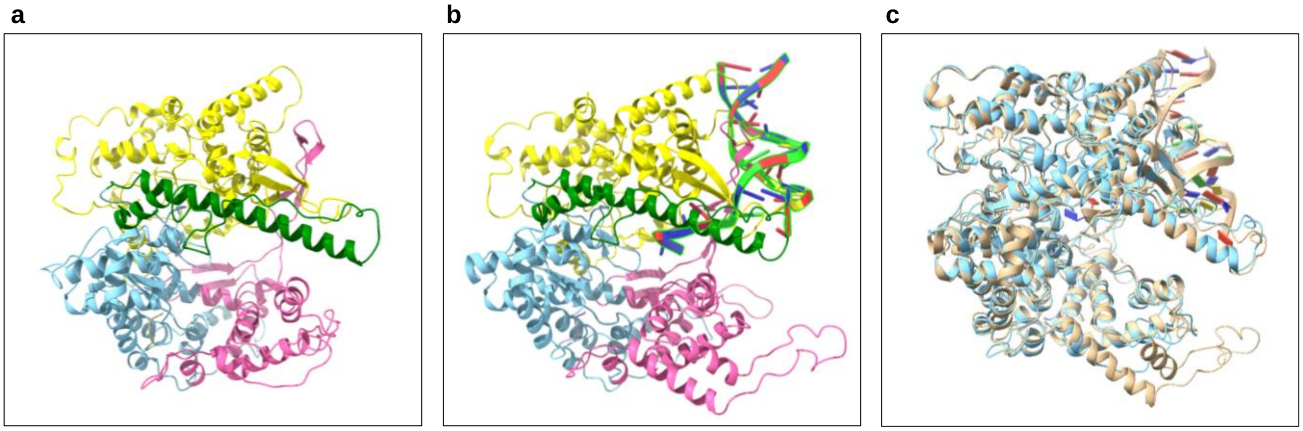

Supplementary Fig. 2 Comparison of predicted EM1 RNAP structure with T7 RNA polymerase.

a Predicted EM1 RNAP structure with Robetta (https://robetta.bakerlab.org/, Baek et al., 2021 [4]).

b Structure of T7 RNA polymerase (PDB ID: 1CEZ [5]). The double helix indicates the T7 promoter DNA sequence.

Three dimensional structures of EM1 RNAP and T7 RNA polymerase contain an N-terminal domain (yellow) and three sub-domains *viz.* thumb (green), palm (sky blue) and finger (hot pink) (annotated according to Borkotoky et al., 2018 [6]).

c Comparison of predicted EM1 RNAP structure (sky blue) with T7 RNA polymerase (gold). The images of models and the structure match were derived with UCSF Chimera X [7].

a

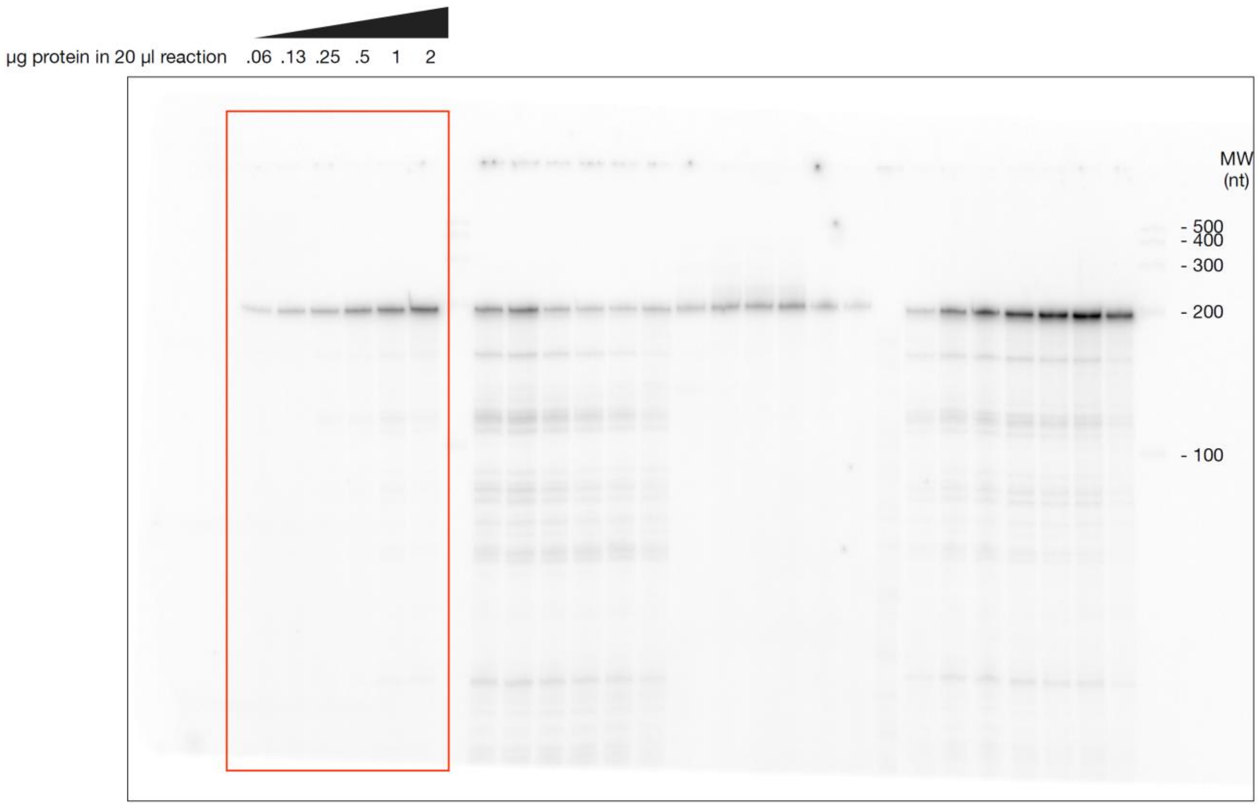
µg protein in 20 µl reaction
.06 .13 .25 .5 1 2
MW
(nt)
- 500
- 400
- 300
- 200
- 100

b

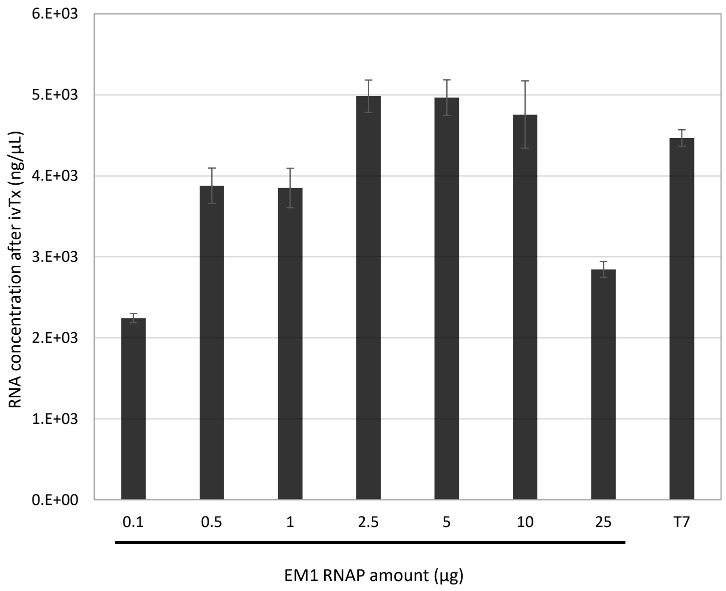
6.E+03
5.E+03
4.E+03
3.E+03
2.E+03
1.E+03
0.E+00
RNA concentration after ivTx (ng/µL)
0.1
0.5
1
2.5
5
10
25
T7
EM1 RNAP amount (µg)

Supplementary Fig. 3 Dose-dependency of EM1 RNAP for *in vitro* transcription.

a Dose-dependency experiment testing EM1 RNAP transcription of 200 nt radio-labelled RNAs in 20 µL reactions incubated for 5 min at at 37°C. Transcripts were resolved on a denaturing gel and detected by phosphor-imagery. Only the lanes within the red rectangle are related to this experiment.

b mRNA concentrations after *in vitro* transcription (ivTx) with different amount of EM1 RNAP in 50 µL reactions. PET2 DNA sequence was amplified with the primer harboring ORF45 promoter and used as template for *in vitro* transcription with different amount of EM1 RNAP at 37°C. As a control, PET2's mRNA was also *in vitro* transcribed at 37°C with T7 RNA polymerase (56 unit, Thermo Scientific) using PET2 DNA sequence with T7 promoter as template. mRNA concentration was quantified with Nanodrop 2000 spectrophotometer. The reaction mixtures without RNA polymerase or without DNA template were used as negative controls and the mRNA concentrations after *in vitro* transcription were below 5 ng/µL. Error bars indicate the standard deviation from three independent experiments.

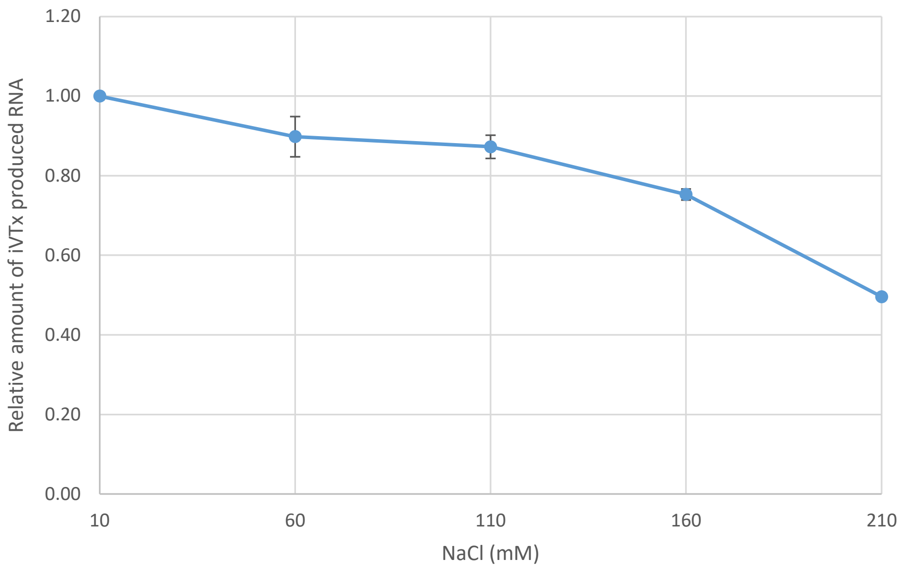

Supplementary Fig. 4 Salt tolerance of EM1 RNAP.

PET2 DNA sequence was amplified with the primer harboring ORF45 promoter and used as template for *in vitro* transcription (ivTx) with 5 µg of EM1 RNAP at 37°C. The concentration of NaCl in the reaction buffers are indicated in the figure. The produced RNA concentration was quantified with NanoDrop 2000 spectrophotometer and normalized to the amount of RNA produced in the routine buffer containing 10 mM NaCl. Error bars indicate the standard deviation from three independent experiments.

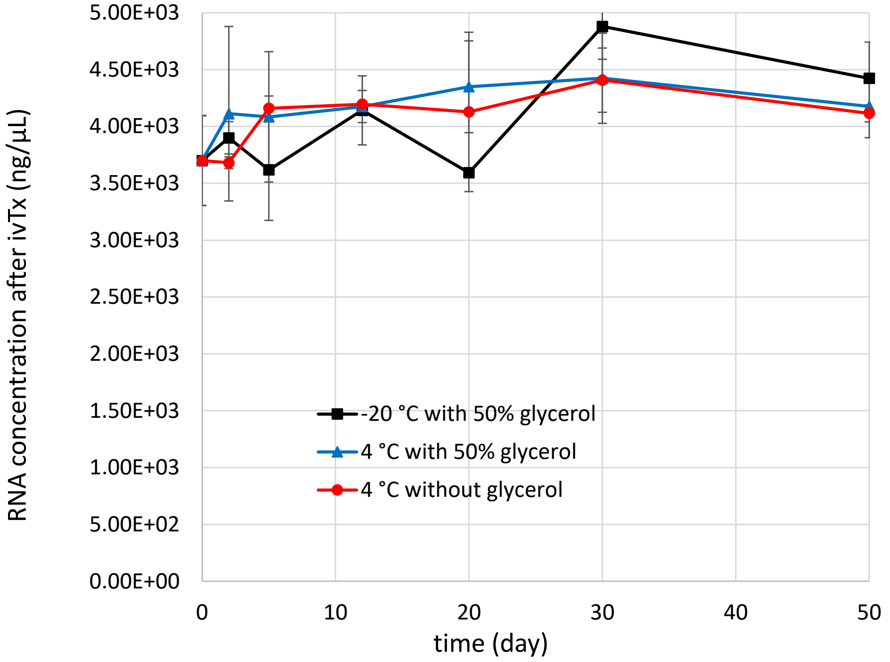

Supplementary Fig. 5 The activities of EM1 RNAP under different storage conditions.

PET2 DNA sequence was amplified with the primer harboring ORF45 promoter and used as template for *in vitro* transcription with 5 µg of EM1 RNAP at 37°C. The EM1 RNAP were stored either with or without glycerol and at 4°C or -20°C. mRNA concentration was quantified with NanoDrop 2000 spectrophotometer. Error bars indicate the standard deviation from three independent experiments.

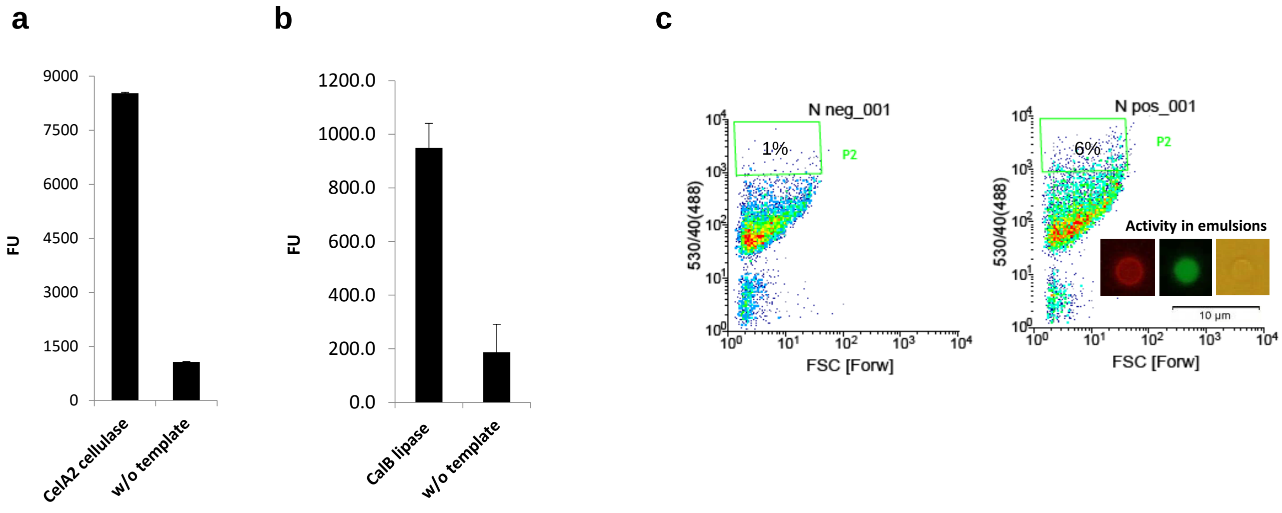

Supplementary Fig. 6 Example proteins produced via our proposed *in vitro* expression system.

PCR-generated gene templates were applied to generate respective mRNAs and subsequently translated using in-house prepared ribosomal cocktail from *E. coli* BL21-CodonPlus (DE3)-RIL cells. **a** An active *in vitro* produced cellulase (CelA2 [8]) with 4-Methylumbelliferyl β-D-cellobioside as substrate. **b** An active *in vitro* produced lipase (CalB [9]) on the substrate methylumbelliferyl caprylate after immobilized on Ni^{2+}-immobilized microtiter plate. FU is fluorescence units quantified using excitation and emission wavelengths of 365 nm and 455 nm, respectively. **c** *in vitro* expressed sfGFP [10] was sorted by FACS with emulsion technology [11] for high throughput screening. Double emulsion vesicles were formed using micro-extruder. Cell-free protein synthesis reaction mixture containing mRNA generated by EM1 RNAP was encapsulated in the inner aqueous phase. Middle and outer phases of the polymersomes were formed from pre-filtered mixtures of 1 mg/ml diblock copolymer poly(ethylene oxide)-b-poly (butadiene) (PEO-b-PBD) in chloroform-hexane (38:62) and 10 % (w/v) polyvinyl alcohol (PVA) in 0.1 M NaCl solution respectively. Vesicle formation was monitored microscopically using bright field (orange) and nile red labeling (red). Polymersomes were then identified and sorted via FACS analysis. Fluorescent microscope (green) and FACS analysis shows successful *in vitro* synthesis of sfGFP in micro-emulsions and screening positive hits thereafter.

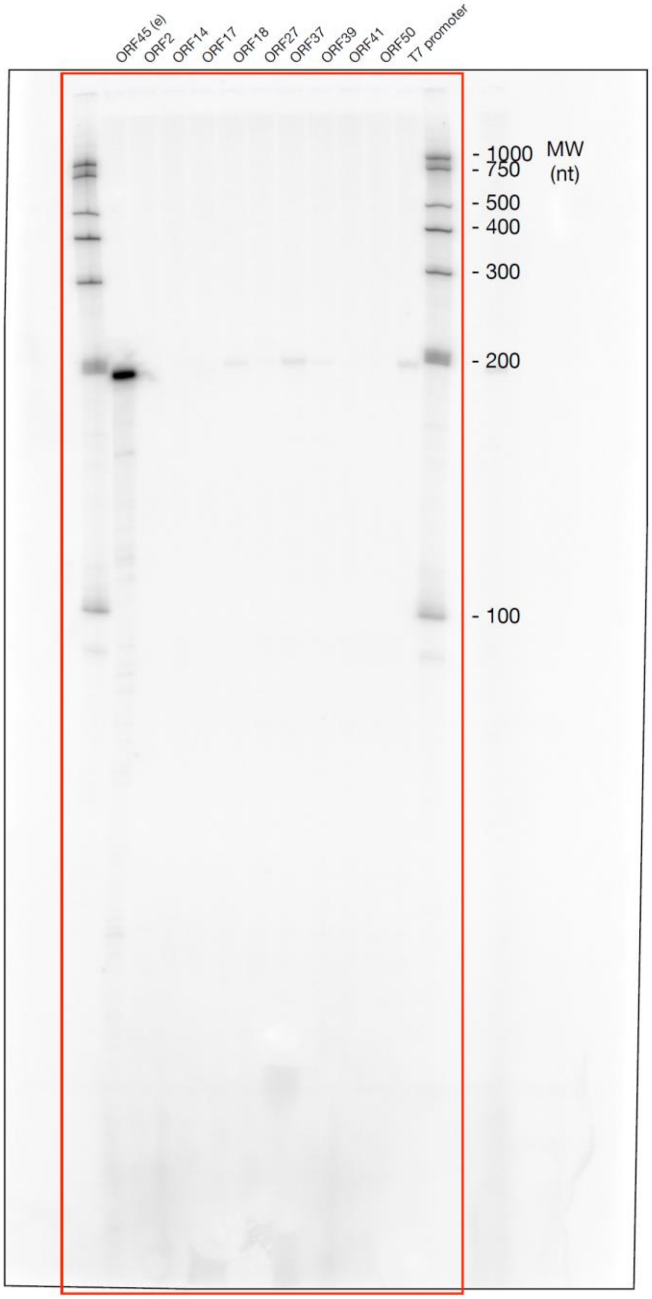

Supplementary Fig. 7 Promoter-dependency experiment testing EM1 RNAP transcription of 200 nt RNAs (original figure of Fig. 3d).

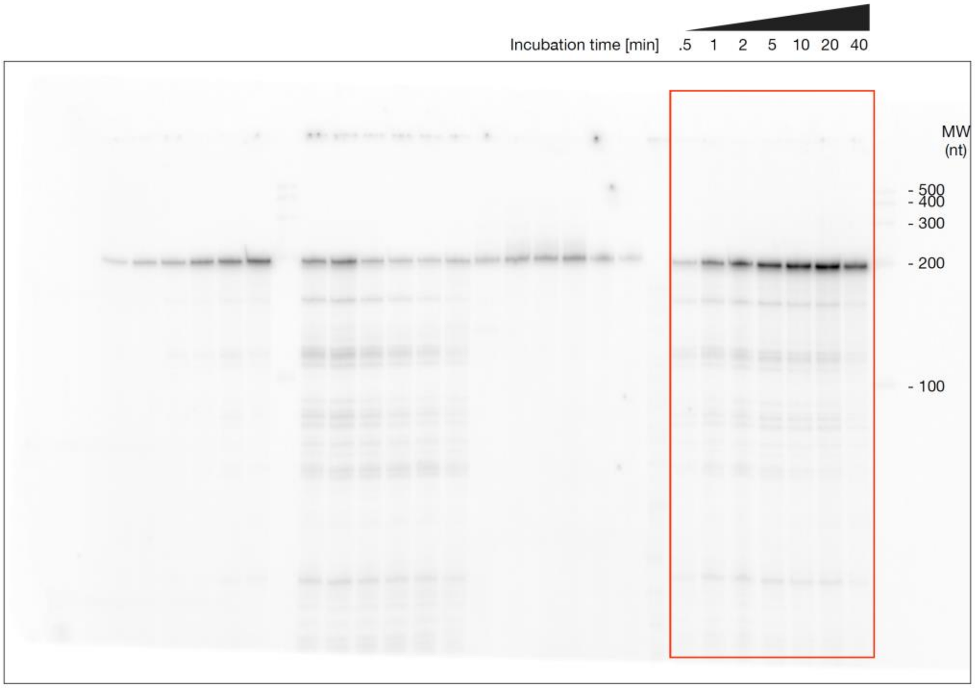

Supplementary Fig. 8 Time course of 200 nt RNA synthesis by EM1 RNAP at 37°C (original figure of Fig. 4a). Only the lanes within the red rectangle are related to this experiment.

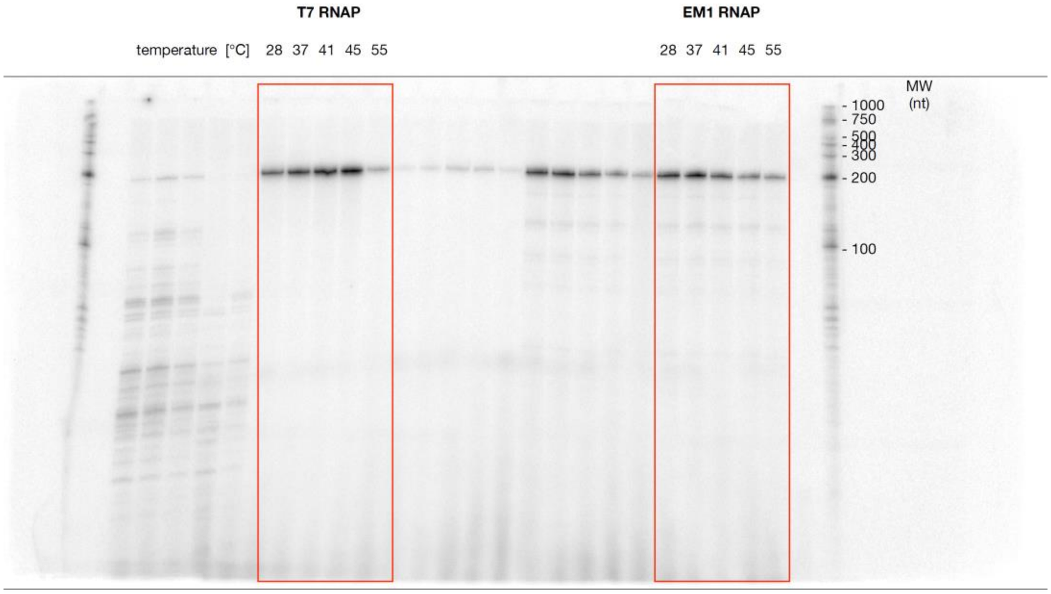

Supplementary Fig. 9 Temperature-dependency experiment testing EM1 RNAP and T7 RNAP transcription of 200 nt RNAs (original figure of Fig. 4c). Only the lanes within the red rectangles are related to this experiment.

a

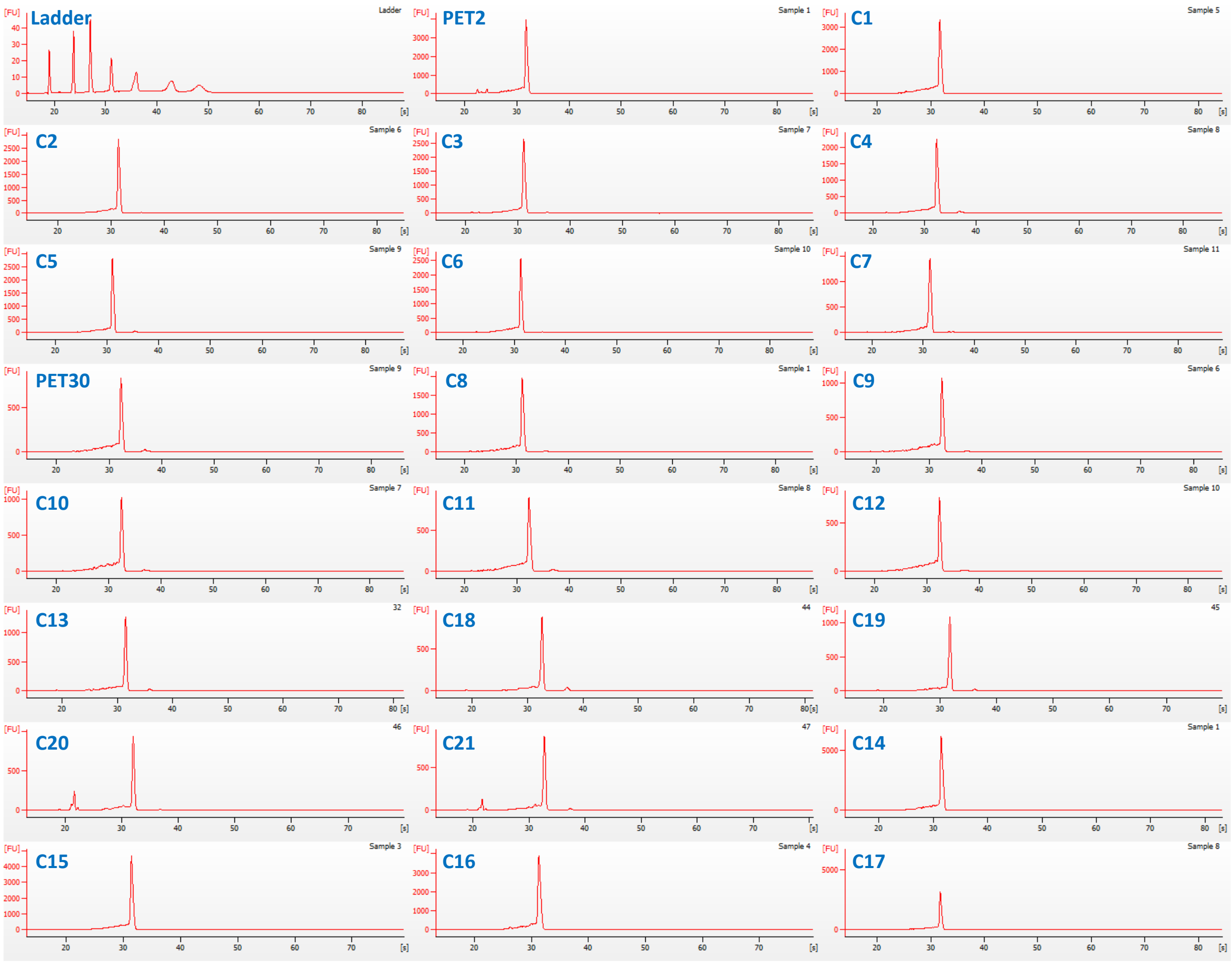
Ladder
PET2
C1
C2
C3
C4
C5
C6
C7
PET30
C8
C9
C10
C11
C12
C13
C18
C19
C20
C21
C14
C15
C16
C17

b

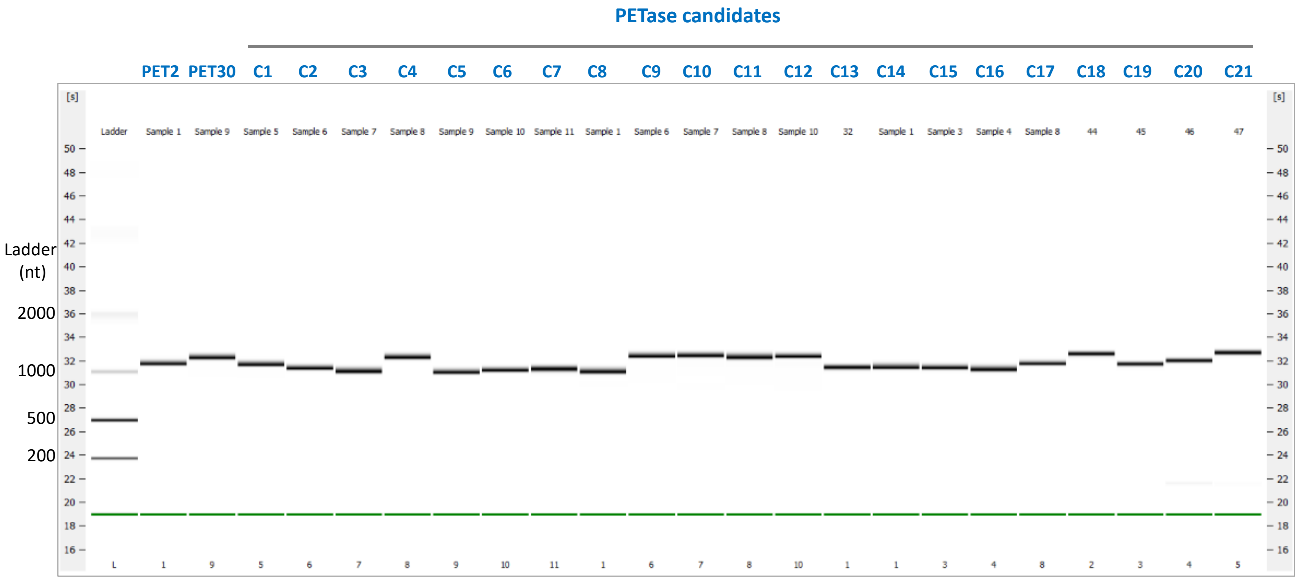

Supplementary Fig. 10 Analyses of *in vitro* produced mRNAs from PETase candidates with EM1 RNAP.

a Original electropherograms of all RNA samples on the Agilent RNA 6000 Pico chips in the Agilent 2100 Bioanalyzer (original data for Fig. 6a).

b Annotated RNA gel figure from electropherograms with Agilent 2100 Expert Software (original figure of Fig. 6a).

2.2 Supplementary Table

Supplementary Table 1 Individual promoters identified by the FIMO search based on ORF45 promoter.

ORF	Predicted Promoter Sequence	Distance to start codon (nt)	FIMO score	FIMO p-value	Predicted function (pBLAST)
**ORF_45**	TCAGAAGTCACACTATAA	18	33.3902	2.58E-11	N-acetyltransferase
**ORF_2**	TCAGAAGGCTTAGCATAG	52	1.43902	0.000209	DNA polymerase I
**ORF_14**	TCAGGGTGCTCTTTAACA	20	-14.0854	0.00886	Phage protein (unknown)
**ORF_27**	TTAGCAGGTATTCTATAA	72	1.17683	0.000283	Terminase large subunit
**ORF_18**	TCTATAGCGACACTAGAT	66	-3.70732	0.000795	Adenylate forming domain-containing protein
**ORF_39**	TGCTCAGTAACAGTGTAA	14	-4.23171	0.00149	GalE / DUF86-containing / flagellar motor switch protein
**ORF_37**	CCAGAGCTTAAAATATAG	9	-4.30488	0.0016	Tail tubular protein
**ORF_17**	GCTGTAGTAACACCCCCA	32	-8.67683	0.00205	Glycoside hydrolase
**ORF_50**	GTAGCACCCGCTCTGTAA	52	-8.89634	0.00278	Exonuclease
**ORF_41**	ACAGCAGCAGCAGGATAT	82	-9.0122	0.00339	Scaffolding protein

References

1. Martino C, *et al.* Protein expression, aggregation, and triggered release from polymersomes as artificial cell-like structures. *Angewandte Chemie (International ed in English)* **51**, 6416-6420 (2012).

2. Mastrobattista E, Taly V, Chanudet E, Treacy P, Kelly BT, Griffiths AD. High-throughput screening of enzyme libraries: *in vitro* evolution of a beta-galactosidase by fluorescence-activated sorting of double emulsions. *Chem Biol* **12**, 1291-1300 (2005).

3. Körfer G, Pitzler C, Vojcic L, Martinez R, Schwaneberg U. *In vitro* flow cytometry-based screening platform for cellulase engineering. *Sci Rep* **6**, 26128 (2016).

4. Baek M, *et al.* Accurate prediction of protein structures and interactions using a three-track neural network. *Science* **373**, 871-876 (2021).

5. Cheetham GMT, Jeruzalmi D, Steitz TA. Structural basis for initiation of transcription from an RNA polymerase–promoter complex. *Nature* **399**, 80-83 (1999).

6. Borkotoky S, Murali A. The highly efficient T7 RNA polymerase: A wonder macromolecule in biological realm. *Int J Biol Macromol* **118**, 49-56 (2018).

7. Pettersen EF, *et al.* UCSF ChimeraX: Structure visualization for researchers, educators, and developers. *Protein Sci* **30**, 70-82 (2021).

8. Ilmberger N, *et al.* Metagenomic cellulases highly tolerant towards the presence of ionic liquids-linking thermostability and halotolerance. *Appl Microbiol Biotechnol* **95**, 135-146 (2012).

9. Uppenberg J, Hansen MT, Patkar S, Jones TA. The sequence, crystal structure determination and refinement of two crystal forms of lipase B from Candida antarctica. *Structure* **2**, 293-308 (1994).

10. Pédelacq JD, Cabantous S, Tran T, Terwilliger TC, Waldo GS. Engineering and characterization of a superfolder green fluorescent protein. *Nat Biotechnol* **24**, 79-88 (2006).

11. Markel U, Essani KD, Besirlioglu V, Schiffels J, Streit WR, Schwaneberg U. Advances in ultrahigh-throughput screening for directed enzyme evolution. *Chem So Rev* **49**, 233-262 (2020).
